# High Migration and Invasion Ability of PGCCs and Their Daughter Cells Associated With the Nuclear Localization of S100A10 Modified by SUMOylation

**DOI:** 10.3389/fcell.2021.696871

**Published:** 2021-07-16

**Authors:** Qi Zhao, Kexin Zhang, Zugui Li, Hao Zhang, Fangmei Fu, Junjie Fu, Minying Zheng, Shiwu Zhang

**Affiliations:** ^1^Department of Pathology, Tianjin Union Medical Center, Nankai University, Tianjin, China; ^2^Graduate School, School of Medicine, Nankai University, Tianjin, China; ^3^3Graduate School, Tianjin University of Traditional Chinese Medicine, Tianjin, China; ^4^Tianjin Medical University, Tianjin, China

**Keywords:** S100A10, polyploidy giant cancer cells, SUMOylation, colorectal cancer, cell infiltration and migration

## Abstract

Our previous studies have confirmed that cobalt chloride (CoCl_2_) or chemoradiotherapy could induce the formation of polyploid tumor giant cells (PGCCs). Polyploid giant cancer cells are a special subpopulation of cancer cells that contribute to solid tumor heterogeneity. The size of PGCC was at least three times larger than regular diploid cancer cells. PGCCs have the properties of cancer stem cells (CSCs) and can express CSC markers CD44 and CD133. Daughter cells derived from PGCCs have strong proliferation, infiltration and migration abilities. However, the detailed molecular mechanism of daughter cells expressing mesenchymal phenotype and displaying strong abilities of proliferation and migration is unclear. As a plasminogen receptor, S100A10 which is closely associated with the invasion and metastasis of malignant tumors, was highly expressed in PGCCs with their daughter cells. In this study, CoCl_2_ was used to induce the formation of PGCCs in LoVo and HCT116 CRC cells. Cell functional experiments, co-immunoprecipitation, MG132 and ginkgolic acid treatment, western blot, and ChIP-Seq were used to identify the mechanism of S100A10 nuclear location. The proliferation and migration abilities of PGCCs and their daughter cells decreased significantly after S100A10 knockdown. In the control cells, S100A10 was mainly ubiquitinated, while in PGCCs and daughter cells, S100A10 was mainly SUMOylated, which was associated with S100A10 nuclear location. After SUMO1 was inhibited, the nuclear S100A10 in PGCCs and daughter cells decreased, and their proliferation and migration abilities significantly decreased. ChIP-Seq combined with real-time fluorescent quantitative PCR showed that S100A10 regulated the expression of neutrophil defensin 3 (*DEFA3*), receptor-type tyrosine-protein phosphatase N2 (*PTPRN2*), and rho guanine nucleotide exchange factor 18 (*ARHGEF18*), which were associated with actin dynamics and cytoskeleton remodeling. The expression of S100A10 in the nuclei and cytoplasm of rectal cancer after neoadjuvant chemoradiation (nCRT) and liver metastases increased compared with that in rectal cancer without nCRT. Taken together, the expression and nuclear localization of S100A10 modified by SUMOylation were associated with the high proliferation and migration of PGCCs and their daughter cells, and the differentiation, metastases, and relapse of CRCs by regulating the expression of *ARHGEF18*, *PTPRN2*, and *DEFA3*.

## Introduction

Surgical treatment is often used to treat malignant solid tumors. However, recurrence and metastasis after surgery is a common cause of death. Colorectal cancer (CRC) is the third most common solid tumor worldwide and its mortality ranks second according to the 2018 statistics (Bray et al., 2018). Despite the new improved treatments, the 5-year survival rate and prognosis in patients with CRC remain unsatisfactory because of the tumor recurrence and metastasis (Siegel et al., 2013; Corcoran et al., 2018).

Our previous studies showed that cobalt chloride (CoCl_2_), a hypoxia mimics, could induce the formation of polyploid giant cancer cells (PGCCs) (Zhang et al., 2013b, 2014; Lopez-Sanchez et al., 2014). Polyploid giant cancer cells are a special subpopulation of cancer cells, which closely associate with the heterogeneity of solid tumors and contribute to cancer chemoresistance (Zhang et al., 2014; Fei et al., 2019c). Polyploid giant cancer cells had the properties of cancer stem cells, and exhibited high resistance to hypoxia and chemotherapeutic drugs (Zhang et al., 2014; Fei et al., 2015, 2019a,b, 2020). Polyploid giant cancer cells could produce small-sized daughter cells via asymmetric cell division. Daughter cells derived from PGCCs expressed epithelial mesenchymal transition (EMT) -related proteins including Twist, N-cadherin, and vimentin (Fei et al., 2015). We have previously reported that irradiation or chemical reagents induced the formation of PGCCs and their daughter cells also displayed strong migration and invasion abilities, which may have been associated with the poor prognosis of patients with colorectal cancer (Fei et al., 2019c). PGCCs could be more frequently observed in high-grade malignancies and metastasis foci than in low-grade tumors and primary sites (Zhang et al., 2006). However, the molecular mechanism of daughter cells derived from PGCCs with strong abilities of infiltration, invasion, and proliferation remains unclear.

Our previous study has confirmed that S100A10 was significantly upregulated in PGCCs with daughter cells after CoCl_2_ treatment compared with the control cells (Zhang et al., 2013a). S100A10 (also known as p11) is a member of the S100 family of EF-hand-type Ca^2+^-binding proteins and participates in various biological processes (Hermann et al., 2012; Donato et al., 2013). The overexpression of S100A10 and S100A2 in CRCs may be used for prognosis assessment in relapse CRCs after adjuvant 5-fluorouracil (5-Fu) therapy and radical surgery (Bresnick et al., 2015). S100A10 usually associates with annexin A2 (ANXA2, p36) to form a heterotetramer complex (AIIt) and is mainly localized to the cell membrane and cytoplasm. AIIt at the extra-cytomembrane exhibits a high affinity for tissue plasminogen activator and promotes the activity of tissue plasminogen activator to cleave plasminogen into plasmin, thereby promoting cancer metastasis (Bresnick et al., 2015). A heterotetramer complex in the cytoplasm can protect S100A10 from ubiquitin-mediated proteasome degradation (He et al., 2008). S100A10 in the cell membrane and cytoplasm plays an important role in the infiltration and invasion of malignant tumors. However, the mechanism of S100A10 nuclear localization in malignant tumors has not been reported. In this study, we confirmed that S100A10 modified by SUMOylation was localized to the nuclei of PGCCs and their daughter cells. The nuclear S100A10 could regulate the expression of neutrophil defensin 3 (*DEFA3*), receptor-type tyrosine-protein phosphatase N2 (*PTPRN2*), and rho guanine nucleotide exchange factor 18 (*ARHGEF18*). These proteins involved in actin dynamics and cytoskeleton remodeling may be associated with EMT and contribute to the proliferation, infiltration and invasion of PGCCs with daughter cells.

## Materials and Methods

### Cell Lines and Induction of PGCCs Formation After CoCl_2_ Treatment

HCT116 and LoVo CRC cells were obtained from the American Type Culture Collection and grown in complete RPMI-1640. Detailed information about CoCl_2_ treatment was described in Supplementary Materials and Methods.

### Western Blots (WB) Analysis

CoCl_2_ treated and untreated cells were collected. Total, cytoplasmic, and nuclear proteins were extracted according to the manufacturer’s instructions (Thermo Fisher Scientific; #SJ252790). The detailed information is provided in the Supplementary Table 1 and Supplementary Materials and Methods.

### Cell Migration and Invasion Assays

The abilities of cell migration and invasion assay were evaluated as previously described (Liu and Zhang, 2020) and detailed information about these assays were provided in the Supplementary Materials and Methods.

### Colony Formation Assay

The detailed information is provided in the Supplementary Materials and Methods.

### Immunocytochemical and Immunohistochemical Staining

The detailed information is provided in the Supplementary Table 1 and Supplementary Materials and Methods.

### Co-immunoprecipitation

Co-IP was used to determine the interactions of SUMO1-S100A10 and ubiquitin-S100A10 in LoVo and HCT116 cells before and after CoCl_2_ treatment. The detailed information is provided in the Supplementary Materials and Methods.

### Cell Viability Assay

The cell viability assay after Ginkgolic acid (GA,15:1) treatment is provided in Supplementary Materials and Methods.

### Ginkgolic Acid (15:1) and MG132 Treatment

MG132 is a proteasome inhibitor that can impair ubiquitin-mediated proteasome degradation. The cells before and after CoCl_2_ treatment were grown in 6-well plates until they reached 80% confluence. MG132 (10 μM, Selleck Chemicals, United States) was added, and cell samples were collected for later experiments after incubation for 6 h. Different concentrations (5, 10, and 20 μM) of GA (15:1, MedChem Express, United States) were added to control cells and PGCCs with daughter cells for 24 h, followed by western blot analysis and other assays.

### 5-fluorouracil and Oxaliplatin Treatment

LoVo and HCT116 cells were treated by 5-fluorouracil (5-Fu) and Oxaliplatin (Oxa) as previously described (Fei et al., 2019c). The detailed information is provided in the Supplementary Materials and Methods.

### Transient siRNA Transfection

The detailed information about transient siRNA transfection is provided in the Supplementary Tables 2, 3, 4, 5 and Supplementary materials and methods.

### Chromatin Immunoprecipitation and Data Analysis

ChIP assays were performed according to the instructions of the Pierce Magnetic ChIP Kit. MEME (Bailey and Elkan, 1994) and DREME (Bailey, 2011) were used to detect the sequence motif, which was used to detect long and short consensus sequences. The position of peak summit around the transcript start sites of genes can predict the interaction sites of proteins and genes. Genes associated with different peaks were identified, and GO and KEGG enrichment analyses were performed. The detailed information is provided in the Supplementary Materials and Methods.

### Real-Time PCR Analysis

The detailed information is provided in the Supplementary Materials and Methods. The sequences of PCR primers are shown in **Supplemental** Table 4.

### Human CRC Samples

Paraffin-embedded human CRC tissue samples (*n* = 218) were obtained from the Department of Pathology of Tianjin Union Medical Center. These tissues were divided into four groups: 55 cases of well-differentiated primary focus (group I), 53 cases of moderately differentiated primary focus (group II), 52 cases of poorly differentiated primary focus (group III), and 58 cases of lymph node metastatic foci (group IV). This study was approved by the Hospital Review Board of Tianjin Union Medical Center, and patient information confidentiality was maintained.

### Scoring of IHC Staining

Protein expression was evaluated as previously described (Liu and Zhang, 2020). The detailed information is provided in the Supplementary Materials and Methods.

### Statistical Analyses

SPSS 22 (SPSS Inc., Chicago, United States) was used to analyze the data in this study. All column diagram data are expressed as means ± SD, and all table data are presented as means ± SEM. The Kruskal-Wallis test was conducted to compare the differences in S100A10-related protein expression in human CRC tissues. Other comparisons were performed using a two-tailed Student’s *t*-test and Pearson chi-square (χ^2^) test. A two-tailed *P* value <0.05 was considered statistically significant.

## Results

### Formation of PGCCs Induced by CoCl_2_ Treatment

**FIGURE 1:**
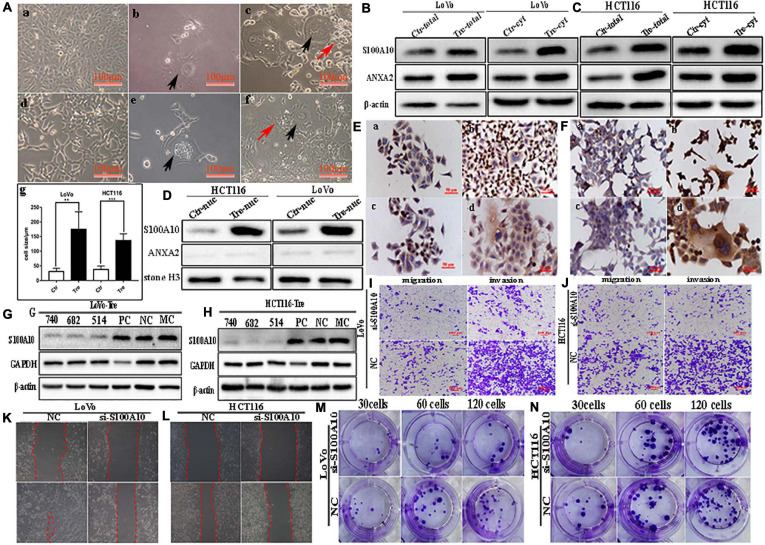
S100A10 and ANXA2 expression in LoVo and HCT116 cells before and after CoCl_2_ treatment. **(A)** Control cells and PGCCs with daughter cells derived from LoVo and HCT116 (100 ×). (a) LoVo control cells. (b) LoVo PGCCs from CoCl_2_ recovered from CoCl_2_ treatment and began to produce daughter cells. (c) LoVo PGCCs and daughter cells. Black arrow indicates PGCC. Red arrow indicates daughter cells. (d) HCT116 control cells. (e) HCT116 PGCCs from CoCl_2_ recovered from CoCl_2_ treatment and began to produce daughter cells. (f) HCT116 PGCCs with daughter cells. Black arrow indicates PGCC. Red arrow indicates daughter cells. (g) Comparison of cell size between control cells and PGCCs. **(B).** The total and cytoplasmic S100A10 and ANXA2 expression in LoVo control cells and PGCCs with daughter cells**. (C).** The total and cytoplasmic S100A10 and ANXA2 expression in HCT116 control cells and PGCCs with daughter cells. **(D)** The nuclear S100A10 and ANXA2 expression in HCT116 and LoVo control cells and PGCCs with daughter cells**. (E)** ICC staining of S100A10 and ANXA2 in LoVo cells before and after CoCl_2_ treatment (ICC, 400×). (a) S100A10 in LoVo control cells (b) S100A10 in LoVo PGCCs with their daughter cells. (c) ANXA2 in LoVo control cells. (d) ANXA2 in LoVo PGCCs with their daughter cells. **(F)** ICC staining of S100A10 and ANXA2 in HCT116 cells before and after CoCl_2_ treatment (ICC, 400×). (a) S100A10 in HCT116 control cells (b) S100A10 in HCT116 PGCCs with their daughter cells. (c) ANXA2 in HCT116 control cells. (d) ANXA2 in HCT116 PGCCs with their daughter cells. **(G)** Total S100A10 expression in LoVo PGCCs with their daughter cells after siRNA S100A10-740, 682, 514, siRNA control and negative control transfection. **(H)** Total S100A10 expression in HCT116 PGCCs with daughter cells after siRNA S100A10-740, 682, 514, siRNA control and negative control transfection. **(I)** The ability of migration and invasion of LoVo PGCCs with daughter cells transfected with siRNA S100A10-740 and siRNA control (100×). **(J)** The ability of migration and invasion of HCT116 PGCCs with daughter cells transfected after siRNA S100A10-740 and siRNA control (100×). **(K)** Wound-healing assay of LoVo PGCCs with daughter cells transfected with siRNA S100A10-740 and siRNA control. **(L)** Wound-healing assay of HCT116 PGCCs with daughter cells transfected with siRNA S100A10-740 and siRNA control. **(M)** Colony formation of LoVo PGCCs with daughter cells transfected with siRNA S100A10-740and siRNA control. **(N)** Colony formation of HCT116 PGCCs with daughter cells transfected with siRNA S100A10-740 and siRNA control. Ctr, control cells; Tre, cells treated with CoCl_2_.

LoVo and HCT116 cells were cultured in T25 flasks to reach 70-80% confluence and treated with 450 μM of CoCl_2_ for 48-72 h (Figure 1A -a, -d). After treatment, most of the small-sized tumor cells died, whereas a few scattered PGCCs survived (Figure 1A -b, -e). The size of PGCCs was significantly larger than that of the control cells (Figure 1A -c, -f, -g). Approximately 10–15 days later, the surviving PGCCs had recovered from CoCl_2_ treatment and generated small-sized daughter cells via asymmetric division. After repeating this treatment 3–4 times, 20-30% of PGCCs and 70–80% of their daughter cells were observed in the flask. The properties of PGCCs and their daughter cells have been well documented in our previous studies (Zhang et al., 2013a, 2014; Fei et al., 2015, 2019b).

### Daughter Cells Derived From PGCCs Have Strong Abilities of Migration, Invasion, and Proliferation

The results of the transwell assay showed that PGCCs with daughter cells gained higher migratory and invasive capacities in comparison with those in the control cells (Supplementary Figures 1A–C). Additionally, the wound-healing assay showed that the migration ability of LoVo and HCT116 PGCCs with daughter cells was significantly increased compared with that of the control cells (Supplementary Figures 1D–F). A plate colony formation assay demonstrated that the proliferation ability in PGCCs with daughter cells was higher than that in the control cells (Supplementary Figures 1G–I).

### S100A10 Is Expressed in Both the Cytoplasm and Nuclei of PGCCs With Daughter Cells

S100A10 was previously reported to be localized in the cytoplasm or membrane. In this study, the expression of S100A10 and ANXA2 was higher in PGCCs and their daughter cells than in the control cells (Figures 1B,C). In the control cells, S100A10 was detected only in the cytoplasm. In PGCCs and their daughter cells, S100A10 was detected in both the cytoplasm (Figures 1B,C and Supplementary Figure 1J -a, -c) and nucleus (Figure 1D and Supplementary Figure 1J -e). ANXA2 was only detected in the cytoplasm (Figures 1B–D, Supplementary Figure 1J -b, -d). The nuclear localization of S100A10 in the PGCCs and their daughter cells was also confirmed using immunocytochemical (ICC) staining (Figures 1E,F). In addition, S100A10 in PGCCs and their daughter cells was knocked down by transient transfection of S100A10-siRNA sequences (740, 682, 514). The inhibition efficiency was evaluated using western blotting (Figures 1G,H). The migration, invasion, and proliferation abilities of PGCCs and their daughter cells were compared before and after S100A10-740i treatment. Figures 1I,J show that the abilities of migration and invasion in cells after S100A10 knockdown (S100A10i) treatment were lower than those in the negative control (NC). The results of the wound-healing assay indicated that the migration ability decreased after S100A10i treatment (Figures 1K,L). Plate cloning assay confirmed that there were fewer clones formed in LoVo and HCT116 PGCCs and their daughter cells after S100A10i than in 30, 60, and 120 NC cells (Figures 1M,N). S100A10 knockdown resulted in significant decrease in invasion, migration, and proliferation abilities of PGCCs and their daughter cells (Supplementary Figure 2A).

### S100A10 Is Modified by SUMOylation in PGCCs and Their Daughter Cells

**FIGURE 2:**
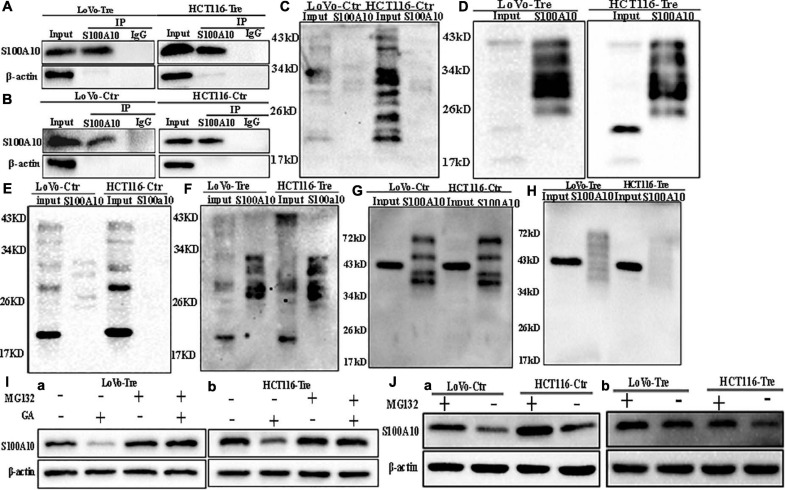
SUMOylation and ubiquitination of S100A10 in LoVo and HCT116 cells before and after CoCl_2_ treatment. **(A)** Results of S100A10 co-immunoprecipitation in LoVo and HCT116 PGCCs with daughter cells (anti-S100A10 was used to perform immunoprecipitation). **(B)** Results of S100A10 co-immunoprecipitation in LoVo and HCT116 control cells (anti-S100A10 was used to perform immunoprecipitation). **(C)** Total lysates of LoVo and HCT116 control cells were immunoprecipitated with anti-S100A10 and immunoblotted with anti-SUMO1. **(D)** Total lysates of LoVo and HCT116 PGCCs with daughter cells were immunoprecipitated with anti-S100A10 and immunoblotted with anti-SUMO1. **(E)** Total lysates of LoVo and HCT116 control cells were immunoprecipitated with anti-S100A10 and immunoblotted with anti-SUMO2/3. **(F)** Total lysates of LoVo and HCT116 PGCCs with daughter cells were immunoprecipitated with anti-S100A10 and immunoblotted with anti-SUMO2/3. **(G)** Total lysates of LoVo and HCT116 control cells were immunoprecipitated with anti-S100A10 and immunoblotted with anti-Ubiquitin (linkage-specific K48). **(H)** Total lysates of LoVo and HCT116 PGCCs with daughter cells were immunoprecipitated with anti-S100A10 and immunoblotted with anti-Ubiquitin (linkage-specific K48). **(I)** (a) S100A10 expression in LoVo PGCCs and daughter cells with and without GA, MG132, and both GA and MG132 treatment. (b) S100A10 expression in HCT116 PGCCs and daughter cells with and without GA treatment, MG132 treatment and the treatment of both GA and MG132, respectively. **(J)** (a) S100A10 expression in LoVo and HCT116 control cells before and after the MG132 treatment. (b) S100A10 expression in LoVo and HCT116 PGCCs with their daughter cells before and after the MG132 treatment. Ctr: control cells; Tre: cells treated with CoCl_2_

S100A10 can be modified by ubiquitination and degraded by the proteasome, which can be inhibited by ANXA2 (Li et al., 2020). Co-immunoprecipitation was used to detect the interaction between ubiquitin, SUMO1, SUMO2/3 and S100A10. The total cell lysates of control and PGCCs with daughter cells were immunoprecipitated with an anti-S100A10 antibody (Figures 2A,B), and then immunoblotted with anti-ubiquitin (linkage-specific K48), anti-SUMO1 and anti-SUMO2/3 antibodies, respectively. There was not interaction between S100A10 and ubiquitin in PGCCs with their daughter cells (Figures 2G,H). MG132 is a proteasome inhibitor that protects ubiquitinated proteins from proteasome-mediated degradation. Ginkgolic acid can inhibit the SUMOylation by interrupting the formation of SUMO-activating enzyme E1-SUMOs thioester complex. After GA treatment, the expression of S100A10 in PGCCs with daughter cells decreased. The decrease of S100A10 in PGCCs with daughter cells was blocked after the treatment of both GA and MG132 (Figure 2I and Supplementary Figure 2D). After treatment with MG132, the expression of S100A10 was detected in both control cells and PGCCs with daughter cells (Figure 2J). In the control cells, the expression level of S100A10 was significantly increased (Figure 2J -a and Supplementary Figure 2C -a). However, there was no significant difference in the expression of S100A10 in PGCCs with daughter cells before and after MG132 treatment (Figure 2J -b and Supplementary Figure 2C -b). These results indicated that S100A10 might not be ubiquitinated in PGCCs and their daughter cells.

**FIGURE 3:**
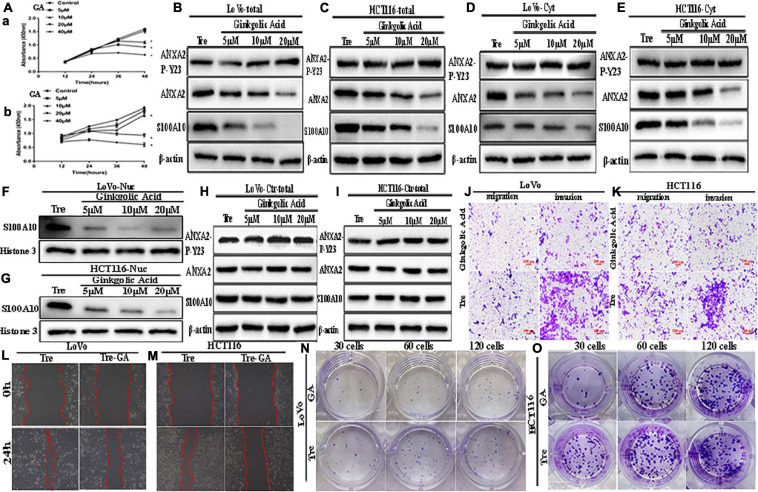
ANXA2-P-Y23, ANXA2, and S100A10 expression in LoVo and HCT116 PGCCs with daughter cells before and after GA treatment and GA inhibits the motility, invasiveness and migration of LoVo and HCT116 PGCCs with daughter cells. **(A)** Effects of GA on the viability of the PGCCs with their daughter cells derived from LoVo and HCT116. Cells treated with 0 μM GA were used as a negative control group. Data are presented as the means ± standard errors. *P < 0.05 treatment vs. control group. (a) LoVo PGCCs with their daughter cells were treated with GA at different concentrations (0, 5, 10, 20, and 40 μM). At each time point (12, 24, 36, and 48 h), cell viability was assessed using CCK8 assay. (b) HCT116 PGCCs with their daughter cells were treated with GA at various concentrations (0, 5, 10, 20, and 40 μM). At each time point (12, 24, 36, and 48 h), cell viability was assessed using CCK8 assay. **(B)** Total ANXA-P-Y23, ANXA2, and S100A10 expression in LoVo PGCCs with their daughter cells after GA treatment at 0, 5, 10, and 20 μM for 24 h. **(C)** Total ANXA-P-Y23, ANXA2, and S100A10 expression in HCT116 PGCCs with their daughter cells after GA treatment at 0, 5, 10, and 20 μM for 24h. **(D)** Cytoplasmic ANXA-P-Y23, ANXA2, and S100A10 expression in LoVo PGCCs with their daughter cells after GA treatment at 0, 5, 10, and 20 μM for 24 h. **(E)** Cytoplasmic ANXA-P-Y23, ANXA2, and S100A10 expression in HCT116 PGCCs with their daughter cells after GA treatment at 0, 5, 10, and 20 μM, respectively, for 24 h. **(F)** Nuclear S100A10 expression in LoVo PGCCs with their daughter cells after GA treatment at 0, 5, 10, and 20 μM for 24 h. **(G)** Nuclear S100A10 expression in HCT116 PGCCs with their daughter cells after GA treatment at 0, 5, 10, and 20 μM, respectively, for 24 h. **(H)** Total ANXA-P-Y23, ANXA2, and S100A10 expression in LoVo control cells after GA treatment at 0, 5, 10, and 20 μM for 24 h. **(I)** Total ANXA-P-Y23, ANXA2, and S100A10 expression in HCT116 control cells after GA treatment at 0, 5, 10, and 20 μM for 24 h. **(J)** The migration and invasion ability of LoVo PGCCs and daughter cells before and after 20 μM of GA treatment for 24 h (100×). **(K)** The migration and invasion ability of HCT116 PGCCs with daughter cells before and after 20 μM of GA treatment for 24 h (100×). **(L)** Wound-healing assay of LoVo PGCCs with daughter cells before and after 20 μM of GA treatment for 24 h. **(M)** Wound-healing assay of HCT116 PGCCs and daughter cells before and after 20 μM of GA treatment for 24 h. **(N)** Colony formation of LoVo PGCCs and daughter cells before and after 20 μM of GA treatment for 24 h. **(O)** Colony formation of HCT116 PGCCs and daughter cells before and after 20 μM of GA treatment for 24 h. Tre, cells treated with CoCl_2_; Cyt, cytoplasm; Nuc, nucleus.

In the PGCCs with daughter cells, the interaction between SUMO1, SUMO2/3, and S100A10 was stronger than it in the control cells (Figures 2C–F). Ginkgolic acid could inhibit SUMOylation *in vitro* and was used to treat control cells and PGCCs with daughter cells. After GA treatment, the expression level of S100A10 was significantly decreased in PGCCs with daughter cells compared to the control cells, which could be eliminated by MG132 treatment (Supplementary Figure 2D -a, -b). There was no change in the expression of S100A10, ANXA2, and ANXA2-P-Y23 in the control cells before and after GA treatment (Figures 3H,I). The results showed that S100A10 was mainly modified by ubiquitin in the control cells, while it was modified by SUMOylation in the PGCCs with daughter cells.

### GA Reduced the Nuclear Expression of S100A10 and Inhibited the Migration, Invasion, and Proliferation of PGCCs With Daughter Cells

To determine whether SUMOylation contributed to the nuclear localization of S100A10, GA was used to treat PGCCs with daughter cells. The cell counting kit-8 (CCK8) assay was performed in PGCCs with daughter cells to determine the appropriate concentration and incubation time (Figure 3A). Our results demonstrated that GA inhibited the proliferation of PGCCs with daughter cells in a time- and dose-dependent manner. Cell viability was seriously impaired at 40 μM for 24 h, and GA at concentrations of 5, 10, and 20 μM was used in this study.

After incubating with different GA concentrations for 24 h, the cells were collected. The total, cytosolic, and nuclear fractions were collected to detect the expression of S100A10, ANXA2, and ANXA2-P-Y23. In the total and cytosolic fractions, the expression of S100A10 and ANXA2 gradually decreased as the increasing concentration of GA treatment (Figures 3B–E and Supplementary Figures 2B -a, -b, -d, -e, 2E -a, -b, -d, -e). The expression of ANXA2-P-Y23 was mildly changed (Figures 3B,C), and the phosphorylated level of ANXA2 was significantly increased (Supplementary Figures 2B -c, -f, 2E -c,-f) in the total and cytosolic fractions. The nuclear localization of S100A10 was inhibited by GA treatment in a dose-dependent manner, indicating that SUMOylation might play an important role in the nuclear localization of S100A10 (Figures 3F,G and Supplementary Figures 2B -g, 2E -g).

To assess the effect of GA on the migration, invasion, and proliferation of PGCCs with daughter cells, cells before and after the treatment with 20 μM GA were used. The results of the transwell assay showed that the number of migratory and invasive cells among the PGCCs with daughter cells treated with GA was lower than that in the untreated cells (Figures 3J,K and Supplementary Figure 2F). The results of the wound-healing assay showed that the scratched areas of the PGCCs with daughter cells before GA treatment were significantly narrower than those in the cells after GA treatment (Figures 3L,M and Supplementary Figure 2G -a). The number of colonies of 30, 60, and 120 GA-treated PGCCs with daughter cells was reduced compared with that in the untreated cells (Figures 3N,O and Supplementary Figure 2G -b).

### SUMO1 Knockdown Inhibits the Nuclear Localization of S100A10 and Decreases the Migration, Invasion, and Proliferation Abilities of PGCCs and Their Daughter Cells

**FIGURE 4:**
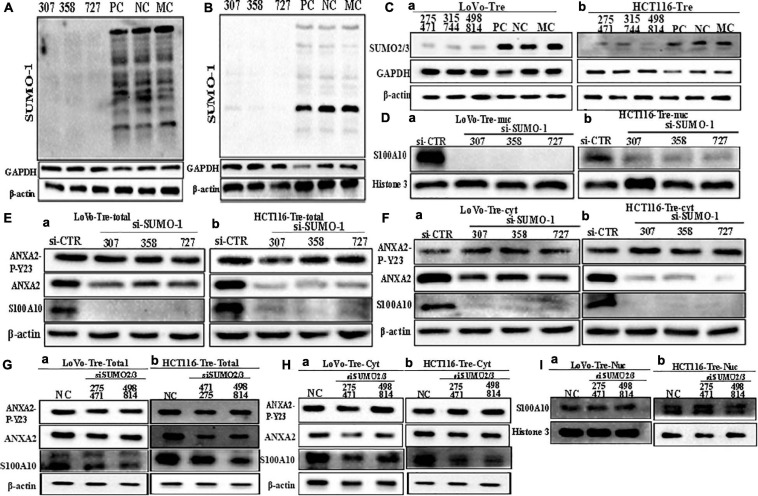
S100A10 and related protein expression in PGCCs with their daughter cells after siRNA SUMO1, siRNA SUMO2/3, siRNA control, and negative control transfection. **(A)** Total SUMO1 expression in LoVo PGCCs with their daughter cells after three sequences of siRNA-SUMO1(307, 358, 727) transfection. **(B)** Total SUMO1 expression in HCT116 PGCCs with daughter cells after three sequences of siRNA-SUMO1(307, 358, 727) transfection. **(C)** (a) Total SUMO2/3 expression in LoVo PGCCs with their daughter cells after three sequences of siRNA-SUMO2/3(275471, 315744, 498814) transfection. (b) Total SUMO2/3 expression in HCT116 PGCCs with daughter cells after three sequences of siRNA-SUMO2/3(275471, 315744, 498814) transfection. **(D).** (a) Nuclear S100A10 expression in LoVo PGCCs with their daughter cells after siRNA-SUMO1 transfection. (b) Nuclear S100A10 expression in HCT116 PGCCs with their daughter cells after siRNA-SUMO1 transfection. **(E)** (a) Total ANXA-P-Y23, ANXA2, and S100A10 expression in LoVo PGCCs with their daughter cells after siRNA-SUMO1 transfection. (b) Total ANXA-P-Y23, ANXA2, and S100A10 expression in HCT116 PGCCs with their daughter cells after siRNA-SUMO1 transfection. **(F)** (a) Cytoplasmic ANXA2-P-Y23, ANXA2, and S100A10 expression in LoVo PGCCs with their daughter cells after siRNA-SUMO1 transfection. (b) Cytoplasmic ANXA2-P-Y23, ANXA2, and S100A10 expression in HCT116 PGCCs with their daughter cells after siRNA-SUMO1 transfection. **(G)** (a) Total ANXA-P-Y23, ANXA2, and S100A10 expression in LoVo PGCCs with their daughter cells after siRNA-SUMO2/3 transfection. (b) Total ANXA-P-Y23, ANXA2, and S100A10 expression in HCT116 PGCCs with their daughter cells after siRNA-SUMO2/3 transfection. **(H)** (a) Cytoplasmic ANXA2-P-Y23, ANXA2, and S100A10 expression in LoVo PGCCs with their daughter cells after siRNA-SUMO2/3 transfection. (b) Cytoplasmic ANXA2-P-Y23, ANXA2, and S100A10 expression in HCT116 PGCCs with their daughter cells after siRNA-SUMO2/3 transfection. **(I)** (a) Nuclear S100A10 expression in LoVo PGCCs with their daughter cells after siRNA-SUMO2/3 transfection. (b) Nuclear S100A10 expression in HCT116 PGCCs with their daughter cells after siRNA-SUMO2/3 transfection. Ctr, control cells; Tre, cells treated with CoCl_2_; Cyt, cytoplasm; Nuc, nucleus.

To further confirm the effects of SUMOylation on the nuclear localization of S100A10, we performed transient transfection by using SUMO1-siRNA sequences (307, 358, 727) and SUMO2/3-siRNA mix sequences (275471, 315744, 498814) to knockdown the expression of SUMO1 and SUMO2/3 in PGCCs with their daughter cells. After transfection, cell samples were immunoblotted with an anti-SUMO1 and anti-SUMO2/3 antibody to detect the transfection effects (Figures 4A–C). The nuclear localization of S100A10 was completely inhibited by SUMO1-siRNA transfection in PGCCs with daughter cells (Figure 4D and Supplementary Figure 3A -c, -f). The total and cytoplasmic expression of S100A10 was declined after SUMO1 knockdown (Figures 4E,F and Supplementary Figure 3A -a, -b, -d, -e). Total and cytoplasmic levels of ANXA2 were moderately downregulated after SUMO1 knockdown, which was also statistically significant (Figures 4E,F and Supplementary Figure 3B -a, -b, -e, -f). The level of phosphorylated ANXA2 at tyrosine 23 did not change and the total ANXA2 level decreased after SUMO1 knockdown (Figures 4E,F and Supplementary Figure 3B -c, -d, -g, -h). According to the results of SUMO2/3-siRNA transfection, we chose sequences 275471 and 498814 for the following experiments (Figure 4C). The total and cytoplasmic expression of S100A10 and ANXA2 in PGCCs with daughter cells after the knockdown SUMO2/3 was mildly decreased, which was statistically significant (Figures 4G,H and Supplementary Figures 3C -a, -b, -d, -e, 3D -a, -b, -d, -e). However, after the knockdown of SUMO2/3, the nuclear expression of S100A10 in PGCCs with daughter cells did not change and the difference had no statistical significance (Figure 4I and Supplementary Figures 3C -g, 3D -g). The total and cytoplasmic level of phosphorylated ANXA2 at tyrosine 23 did not change after SUMO2/3 knockdown (Figures 4G,H and Supplementary Figures 3C -c, -f, 3D -c, -f). In conclusion, S100A10 was modified by SUMO1 not SUMO2/3, which regulated the nuclear location of S100A10.

**FIGURE 5:**
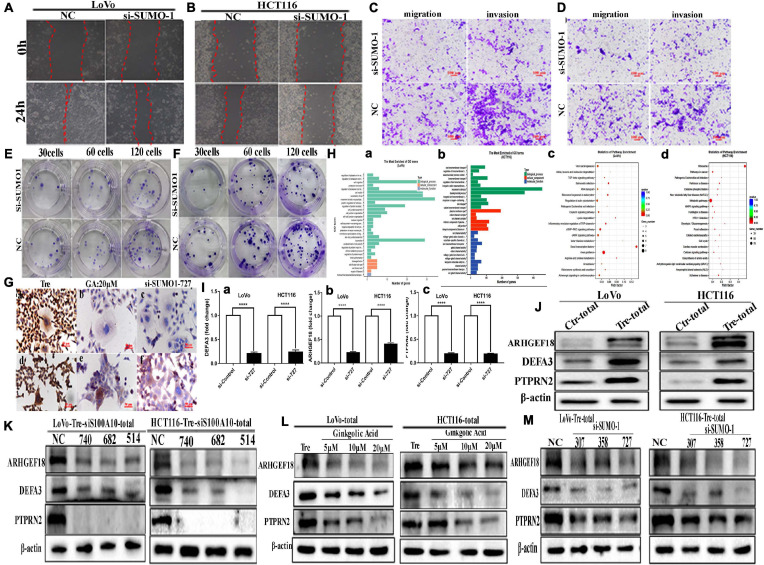
The motility, invasiveness and migration of LoVo and HCT116 PGCCs and daughter cells before and after SUMO1 knockdown and the results of ChIP-Seq and real-time PCR in these cells. **(A)** Wound-healing assay in LoVo PGCCs with their daughter cells transfected with SUMO1-727 and control siRNAs. **(B)** Wound-healing assay in HCT116 PGCCs with their daughter cells transfected with SUMO1-727 and control siRNAs. **(C)** The abilities of migration and invasion in LoVo PGCCs with their daughter cells transfected with SUMO1-727 and control siRNAs (100×). **(D)** The migration and invasion ability in LoVo PGCCs with their daughter cells transfected with SUMO1-727 and control siRNAs (100×). **(E)** Colony formation in LoVo PGCCs with their daughter cells transfected with SUMO1-727 and control siRNAs. **(F)** Colony formation in HCT116 PGCCs with their daughter cells transfected with SUMO1-727 and control siRNAs. **(G)** ICC staining of S100A10 in the LoVo and HCT116 PGCCs with daughter cells (ICC, 400×). (a) ICC staining of S100A10 in LoVo PGCCs with daughter cells. (b) ICC staining of S100A10 in LoVo PGCCs with daughter cells after GA treatment at 20 μM. (c) ICC staining of S100A10 in LoVo PGCCs with daughter cells after SUMO1-siRNA 727 transfection. (d) ICC staining of S100A10 in HCT116 PGCCs with daughter cells. (b) ICC staining of S100A10 in HCT116 PGCCs with daughter cells after GA treatment at 20 μM. (c) ICC staining of S100A10 in HCT116 PGCCs with daughter cells after SUMO1-siRNA 727 transfection. **(H)** (a) GO functional enrichment of targets associated with S100A10 in the PGCCs with daughter cells of LoVo ChIP-seq peaks for biological process. (b) GO functional enrichment of targets associated with S100A10 in the PGCCs with daughter cells of HCT116 ChIP-seq peaks for biological process. (c) KEGG pathway clustering analysis of targets associated with S100A10 in the PGCCs with daughter cells of LoVo ChIP-seq peaks. (d) KEGG pathway clustering analysis of targets associated with S100A10 in the PGCCs with daughter cells of HCT116 ChIP-seq peaks. **(I)** Immunoprecipitated DNA before and after the knockdown of SUMO1 was analyzed using specific primers of *DEFA3*, *ARHGEF18*, and *PTPRN2*. (a) *DEFA3*, (b) *ARHGEF18*, (c) *PTPRN2*. **(J)** The total ARHGEF18, DEFA3, and PTPRN2 expression in LoVo and HCT116 cells before and after CoCl_2_ treatment. **(K)** Total ARHGEF18, DEFA3, and PTPRN2 expression in LoVo PGCCs with daughter cells after transfection with S100A10-740, 682, 514, siRNA control, and negative control transfection. **(L)** Total ARHGEF18, DEFA3, and PTPRN2 expression in LoVo and HCT116 PGCCs with their daughter cells after GA treatment at 0, 5, 10, and 20 μM for 24 h. **(M)** Total ARHGEF18, DEFA3, and PTPRN2 expression in LoVo and HCT116 PGCCs with their daughter cells after siRNA SUMO1-207, 358, 727, siRNA control, and negative control transfection.

Inhibition of SUMO1 expression also decreased the migration, invasion, and proliferation of PGCCs with daughter cells. In the wound-healing assay, the wound spaces in the NC group were smaller than those of the SUMO1 knockdown (Figures 5A,B and Supplementary Figure 3E- a). The results of the transwell assay further demonstrated that the migration and invasion abilities were decreased after SUMO1 knockdown (Figures 5C,D and Supplementary Figures 3E -b, 3F -a). The colony formation efficiency after SUMO1 knockdown was significantly lower than that in the NC group (Figures 5E,F and Supplementary Figure 3F -b).

ICC staining of S100A10 further demonstrated that SUMOylation was involved in the nuclear localization of S100A10. In PGCCs with daughter cells, the expression of S100A10 was observed in both the cytoplasm and nucleus (Figure 5G -a, -d). When PGCCs with daughter cells were treated with 20 μM of GA or transfected with SUMO1 siRNA, the expression of S100A10 was only observed in the cytoplasm (Figure 5G -b, -c, -e, -f).

### The Nuclear Localization of S100A10 Regulates the Expression of *DEFA3*, *PTPRN2*, and *ARGHEF18*

Chromatin immunoprecipitation (ChIP) assay was performed using an antibody against S100A10, followed by sequencing to assess the potential targets of S100A10. The results showed that 4148 significant ChIP-seq peaked in HCT116-derived PGCCs with daughter cells, and 1380 peaked in LoVo-derived PGCCs with daughter cells. Genes with overlapping peaks were then enriched by Gene Ontology [GO], which revealed that the downstream target genes of S100A10 participated in various processes including cell migration and motility (Figure 5H -a, -b). Moreover, Kyoto Encyclopedia of Genes and Genomes (KEGG) pathway analysis also revealed that diverse tumor-related pathways were associated with S100A10, including the MAPK signaling pathway, TGF- beta signaling pathway and so on (Figure 5H -c, -d). The target genes were further screened through the analysis of the narrow peaks in the two cell lines according to the -log10q value. According to the gene function, *DEFA3*, *PTPRN2*, and *ARHGEF18* were selected, which are associated with the regulation of cytoskeleton dynamics and cell migration. To detect whether S100A10 actually binds to the *DEFA3, PTPRN2*, and *ARHGEF18*, a ChIP assay of S100A10 was performed in PGCCs with daughter cells before and after siRNA SUMO1 transfection. DNA samples for the ChIP assay isolated from the PGCCs wand their daughter cells before and after SUMO1 knockdown were analyzed using real-time PCR. The results showed that all the levels of immunoprecipitated DNA of *DEFA3*, *PTPRN2*, and *ARHGEF18* bound to S100A10 were decreased after SUMO1 knockdown (Figure 5I). The protein levels of ARHGEF18, DEFA3, and PTPRN2 were upregulated in PGCCs with daughter cells (Figure 5J and Supplementary Figure 3G).

The total protein expression levels of ARHGEF18, DEFA3, and PTPRN2 were decreased after S100A10i treatment (Figure 5K and Supplementary Figures 4A,B). To further study the effect of SUMOylated S100A10 on the expression of ARHGEF18, DEFA3, and PTPRN2, PGCCs with daughter cells were treated with 0, 5, 10, and 20 μM of GA and the total expression levels of ARHGEF18, DEFA3, and PTPRN2 were gradually decreased in a concentration-dependent manner (Figure 5L and Supplementary Figure 4C). The total expression of ARHGEF18, DEFA3, and PTPRN2 was also decreased when S100A10 expression was decreased after SUMO1 knockdown (Figure 5M and Supplementary Figure 4D).

### 5-fluorouracil and Oxaliplatin Treatment Promotes the Nuclear Localization of S100A10 by SUMOylation

**FIGURE 6:**
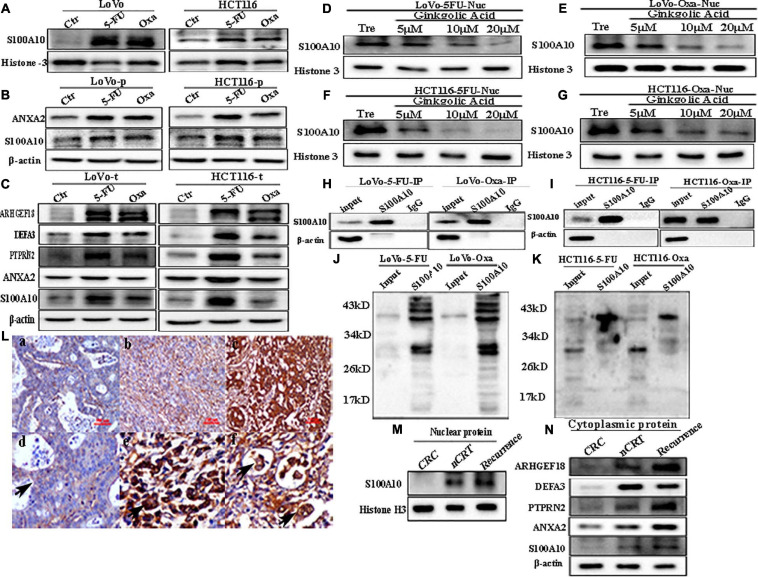
**(A)** Nuclear S100A10 expression in LoVo and HCT116 cells before and after treatment with 5-Fu and Oxa, respectively. **(B)** Cytoplasmic ANXA2 and S100A10 expression in LoVo and HCT116 cells before and after treatment with 5-Fu and Oxa, respectively. **(C)** Total ANXA2, S100A10, ARHGEF18, DEFA3, and PTPRN2 expression in LoVo and HCT116 cells before and after treatment with 5-Fu and Oxa, respectively. **(D)** Nuclear S100A10 expression in 5-Fu-treated LoVo cells after GA treatment at 0, 5, 10, and 20 μM for 24 h. **(E)** Nuclear S100A10 expression in Oxa-treated LoVo cells after GA treatment at 0, 5, 10, and 20 μM for 24 h. **(F)** Nuclear S100A10 expression in 5-Fu-treated HCT116 cells after GA treatment at 0, 5, 10, and 20 μM for 24 h. **(G)** Nuclear S100A10 expression in Oxa-treated HCT116 cells after GA treatment at 0, 5, 10, and 20 μM for 24 h. **(H)** Results of S100A10 co-immunoprecipitation in LoVo cells after treatment with 5-Fu and Oxa, respectively. **(I)** Results of S100A10 co-immunoprecipitation in HCT116 cells after treatment with 5-Fu and Oxa, respectively. **(J)** Total lysates of LoVo cells after treatment with 5-Fu and Oxa were immunoprecipitated with anti-S100A10 and immunoblotted with anti-SUMO1, respectively. **(K)** Total lysates of HCT116 cells after treatment with 5-Fu and Oxa were immunoprecipitated with anti-S100A10 and immunoblotted with anti-SUMO1, respectively. **(L)** IHC staining of S100A10 in the human CRC tissues. (a) S100A10 expression in the well-differentiated CRC tissue (IHC, 200×). (b) S100A10 expression in the moderately differentiated CRC tissue (IHC, 200×). (c) S100A10 expression in the poorly differentiated CRC tissue (IHC, 200×). (d) Cytoplasmic expression of S100A10 in CRC cells (Black arrow heads, IHC, 400×). (e) Nuclear staining in cancer cells expressing S100A10 in CRC tissues (Black arrow heads, IHC, 400×). (d) Cytoplasmic and nuclear expression of S100A10 in PGCCs of CRC tissues (Black arrows head, IHC, 400×). **(M)** Nuclear expression of S100A10 in samples from patients (radical operation) treated with neoadjuvant chemoradiation (nCRT), those not treated with nCRT (CRC) and recurrent liver metastases. **(N)** Cytoplasmic expression of ANXA2, S100A10, ARHGEF18, DEFA3, and PTPRN2 in samples from patients (radical operation) treated with neoadjuvant chemoradiation (nCRT), those not treated with nCRT (CRC) and those with recurrence.

To study the effect of chemotherapeutic drug treatment on the expression and subcellular localization of S100A10 in CRC cells, LoVo and HCT116 were treated with 5-Fu and Oxa for three times. The nuclear expression level of S100A10 increased after 5-Fu and Oxa treatment (Figure 6A and Supplementary Figure 4E -c). The total and cytoplasmic expression of ANXA2 and S100A10 was increased after the treatment with 5-Fu and Oxa (Figures 6B,C and Supplementary Figure 4E -a, -b, -d, -e). The nuclear-located S100A10 in 5-Fu or Oxa -treated LoVo and HCT116 cells could also be inhibited by GA in a dose-dependent manner (Figures 6D–G and Supplementary Figure 4F). The total cell lysates of the 5-Fu- and Oxa-treated cells were immunoprecipitated with an anti-S100A10 antibody (Figures 6H,I) and immunoblotted with an anti-SUMO1 antibody (Figures 6J,K). The results showed that S100A10 was modified by SUMOylation after 5-Fu and Oxa treatment. The expression levels of ARHGEF18, DEFA3 and PTPRN2 were upregulated after 5-Fu and Oxa treatment (Figure 6C and Supplementary Figure 4G).

### Expression of S100A10-Related Protein in Human CRC Tissues

Human CRC tissues were used to evaluate the association between S100A10 expression level, tumor differentiation, and lymph node metastatic foci (Figure 6L, -a, -b, c). The differences in S100A10 staining index among the four groups were statistically significant (*P* = 0.000, Table 1). In addition, the expression of S100A10 was mainly distributed in the cytoplasm of tumor cells in group I (Figure 6L, -d). In group III, S100A10 expression was located in both the cytoplasm and nucleus of cancer cells (Figure 6L, -e). The staining index of S100A10 nuclear expression was significantly higher in poorly differentiated CRC than in well-differentiated CRC (*P* = 0.000) and moderately differentiated CRC (*P* = 0.018). The staining index of S100A10 nuclear expression was also higher in lymph node metastatic foci than in well-differentiated CRC (*P* = 0.000) and moderately differentiated CRC (*P* = 0.003) (Table 2). S100A10 was localized in both the cytoplasm and nucleus of PGCCs (Figure 6K -f).

**TABLE 1:** Differences of S100A10 staining index in different group of human CRCs.

	**group**	**n**	**Staining index for S100A10**	**Value of statistics**	***P***
Well-differentiated CRCs	group I	55	2.800 ± 1.967	χ^2^ = 51.973	0.000*
Moderately differentiated CRCs	group II	53	3.641 ± 2.207		
Poorly differentiated CRCs	group III	52	6.442 ± 1.895		
Lymph node metastatic foci	group IV	58	5.645 ± 2.076		

*^∗^*P* < 0.05: statistically significant. (*P*: difference among the three groups; *P*1 (difference between groups I and II) = 0.002; *P*2 (difference between groups II and III) = 0.000; *P*3 (difference between groups III and IV) = 0.593; *P*4 (difference between groups I and IV) < 0.000; *P*5 (difference between groups II and IV) = 0.000. CRCs: colorectal cancers.*

**TABLE 2:** Comparison of staining index of S100A10 nuclear expression in human CRC tissues.

	**group**	**n**	**Staining index for S100A10**	**Value of statistics**	***P***
Well-differentiated CRCs	group I	55	1.000 ± 0.000	χ^2^ = 44.108	0.000*
Moderately differentiated CRCs	group II	53	1.377 ± 0.713		
Poorly differentiated CRCs	group III	52	1.712 ± 0.824		
Lymph node metastatic foci	group IV	58	1.827 ± 0.861		

*^∗^*P* < 0.05: statistically significant. (*P*: difference among the four groups; *P*1 (difference between groups I and II) = 0.000; *P*2 (difference between groups II and III) = 0.018; *P*3 (difference between groups III and IV) = 0.485; *P*4 (difference between groups I and IV) = 0.000; *P*5 (difference between groups II and IV) = 0.003. CRC: colorectal cancer.*

Human moderately differentiated rectal cancer tissues from patients treated with and without neoadjuvant chemoradiation (nCRT), and with recurrent liver metastases were collected to compare the expression and subcellular location of S100A10. The expression of S100A10 was increased in both the cytoplasm and nucleus of nCRT-treated and recurrent liver metastases tissues compared with those of moderately differentiated samples without nCRT (Figures 6M,N and Supplementary Figure 4G -a, -c). The expression levels of ANXA2, DEFA3, ARHGEF18, and PTPRN2 were also upregulated in the cytoplasm of CRCs after nCRT and recurrence of liver metastases (Figure 6N and Supplementary Figure 4G, -b, -d, -e, -f).

## Discussion

CoCl_2_ and chemoradiotherapy can induce the formation of PGCCs, and daughter cells derived from these PGCCs have strong abilities of proliferation, infiltration, and invasion. Studies have demonstrated that PGCCs with daughter cells could exert important influences on the progression of malignant tumors, including metastasis, chemoresistance, and tumor relapse (Lv et al., 2014; Niu et al., 2017; Wang et al., 2017, 2019; Mirzayans et al., 2018; Xuan et al., 2018; Arun et al., 2019; Chen et al., 2019; Bharadwaj and Mandal, 2020). Here, we demonstrated that S100A10 was involved in regulating the migration and invasion of PGCCs with daughter cells in CRC cells.

The membrane-localized S100A10 promotes cancer invasion by regulating tissue plasminogen activator activity and plasmin generation (Bresnick et al., 2015). S100A10 is unable to exist in the absence of ANXA2 and is promptly degraded when cytoplasmic ANXA2 is depleted (Puisieux et al., 1996; He et al., 2008). S100A10 can be degraded by proteasomes. Lu et al. reported that S100A10 and ANXA2 were both observed in the nucleus of breast cancer stem cells after paclitaxel treatment (Lu et al., 2020). However, the mechanism by which S100A10 and ANXA2 are transported into the nucleus has not been reported. Under hypoxic conditions, the levels of SUMOylated proteins increase in cancer cells, including those modified with SUMO-1 (Shao et al., 2004). SUMOylation is a prevalent post-translation modification that occurs at the lysine residue and can regulate the response of cells to stress (Guo and Henley, 2014). In this study, CoCl_2_ was used to induce the formation of PGCCs and their daughter cells. The expression levels of S100A10 and ANXA2 were increased in the total and cytoplasmic fractions after CoCl_2_ treatment. S100A10 and ANXA2 were mainly expressed in the cytoplasm of control cells. However, the results of WB and ICC showed that S100A10 was also detected in the nuclear fractions of PGCCs and their daughter cells, while ANXA2 was not detected in the nucleus.

Results of S100A10 Co-immunoprecipitation showed that ubiquitin-modified S100A10 was expressed in the control cells, and SUMOylated S100A10 appeared in the PGCCs with daughter cells, which confirmed that S100A10 was mainly modified by SUMOylation after CoCl_2_ treatment. This result was further confirmed by the treatment with MG132 and GA. The expression of S100A10 increased in the control cells, and there was no change in S100A10 expression in the PGCCs with daughter cells after MG132 treatment. After GA treatment, the nuclear expression of S100A10 was gradually decreased, which indicated that SUMOylation directly mediated the nuclear localization of S100A10. Furthermore, the total and cytoplasmic expression of S100A10 was decreased in PGCCs with daughter cells, which showed that S100A10 was re-ubiquitinated because the lysine site became accessible after SUMOylation was inhibited by GA treatment. S100A10 expression increased in PGCCs with daughter cells treated with both MG132 and GA compared with the cells treated with GA alone, indicating that re-ubiquitin of S100A10 could not be degraded because of the use of MG132. The formation of AIIt in the cytoplasm can protect S100A10 from ubiquitin-dependent proteasome degradation, and the phosphorylation of tyrosine 23 can promote the formation of the complex. The expression of ANXA2 was also decreased after GA treatment.

There are four subunits of SUMOylation: SUMO1, SUMO2, SUMO3, and SUMO4 (Seeler and Dejean, 2017). SUMO1, SUMO2, and SUMO3 have similar three-dimensional structures. SUMO1 mainly participates in normal cellular physiology and the expression of SUMO1 was upregulated under hypoxic condition (Comerford et al., 2003; Shao et al., 2004), whereas SUMO2 and SUMO3 are mainly associated with the cell stress response. SUMO4, another SUMO paralog, shares 86% sequence homology with SUMO-2 and SUMO-3, however, its function remains enigmatic as it may be non-conjugated under normal physiological conditions (Wilkinson and Henley, 2010; Flotho and Melchior, 2013). To further clarify which SUMO subunits modulate the nuclear location of S100A10 after CoCl_2_ treatment, we knocked down the expression of SUMO1 and SUMO2/3, respectively. The results showed that the nuclear expression of S100A10 was blocked in PGCCs with daughter cells as well as in the cytoplasm after SUMO1 knockdown. However, the nuclear expression of S100A10 did not change and the cytoplasmic expression was mildly decreased. It is reasonable to say that S100A10 was modified by SUMO1 to translocate into the nucleus. GA treatment and SUMO1 knockdown inhibited the migration, invasion, and proliferation of PGCCs with daughter cells. MG132 can inhibit ubiquitin-mediated proteasome degradation, and the expression level of S100A10 should be increased after MG132 treatment. SUMOylation can stabilize the expression of S100A10 by competing with ubiquitination and facilitating the nuclear localization of S100A10. The results showed that the inhibition of S100A10 SUMOylation could impair the migration, invasion, and proliferation of PGCCs with daughter cells. Based on these results, we can conclude that S100A10 was modified by SUMOylation in PGCCs with daughter cells and by ubiquitination in control cells. The nuclear localization of S100A10 is regulated by SUMOylation.

The mechanisms of S100A10 nuclear location promoting metastasis, invasion and proliferation in PGCCs with daughter cells were also studied. Result of the ChIP-Seq assay combined with GO and KEGG enrichment analysis indicated that S100A10 in the nuclear associated with the expression of *ARHGEF18*, *PTPRN2*, and *DEFA3*, which related to actin dynamics and cytoskeleton remodeling (Nagata and Inagaki, 2005; Gunes et al., 2013). ARHGEF18 is a Rho-guanine nucleotide exchange factor. ARHGEF18 combined with Sept9b can regulate the stress fibers to modulate cell shape changes. Small GTPase Rho and septin family proteins are thought to be associated with tumorigenesis, and ARHGEF18 provides a molecular link between septins and Rho signaling (Nagata and Inagaki, 2005). As a member of the protein tyrosine phosphatase family, PTPRN2 also promoted breast cancer cell migration by regulating actin dynamics through the dephosphorylation of phosphatidylinositol 4,5-bisphosphate. Overexpression of PTPRN2 is associated with worse overall survival and distal metastasis-free survival in patients with breast cancer (Sengelaub et al., 2016). DEFA3 belongs to the α-defensins secreted by neurophils, and the preoperative plasma levels of DEFA3 are positively associated with the progression and pathological stages of bladder cancer (Gunes et al., 2013). DEFA3 was upregulated in tumor tissue compared with the normal colonic mucosa and negatively correlated with the prognosis in patients with CRC (Albrethsen et al., 2006). Results of the real-time PCR confirmed that the DNA levels of *ARHGEF18*, *PTPRN2*, and *DEFA3* bound to S100A10 were decreased after SUMO1 knockdown in the PGCCs with daughter cells. The downregulation of ARHGEF18, PTPRN2, and DEFA3 in the PGCCs with daughter cells after S100A10 knockdown indicated that S100A10 served as an upstream factor of these three proteins. Inhibition of SUMOylation in PGCCs with daughter cells either by GA treatment or SUMO1 silencing could also inhibit the expression of ARHGEF18, PTPRN2, and DEFA3, which further confirmed that the expression of these proteins can be inhibited after SUMOylated S100A10 inhibition. The expression level of nuclear S100A10 associated with the expression of *DEFA3*, *PTPRN2*, and *ARHGEF18*, and regulated the proliferation, infiltration and invasion of PGCCs with daughter cells by the process of EMT.

We previously reported that irradiation and treatment with chemical reagents including 5-Fu and Oxa could also induce the formation of PGCCs, and this kind of PGCCs could also generate daughter cells with strong invasion and migration abilities (Fei et al., 2019c). S100A10 was modified by SUMO1 and the expression increased in both the cytoplasm and nucleus in the PGCCs with daughter cells after 5-Fu and Oxa treatment. The expression levels of ARHGEF18, PTPRN2, and DEFA3 were also increased after 5-Fu and Oxa treatment. In human CRC tissues, the expression level of S100A10 was positively correlated with tumor differentiation, and the positive rate of S100A10 nuclear localization was the highest in the poorly differentiated CRCs compared with that in well and moderately differential CRC. In addition, the expression of S100A10 in the nuclei and cytoplasm of rectal cancer after nCRT and liver metastatic foci increased compared with that in rectal cancer without nCRT, indicating that SUMO-modified S100A10 may be a new target for CRC clinical therapy.

## Conclusion

**FIGURE 7:**
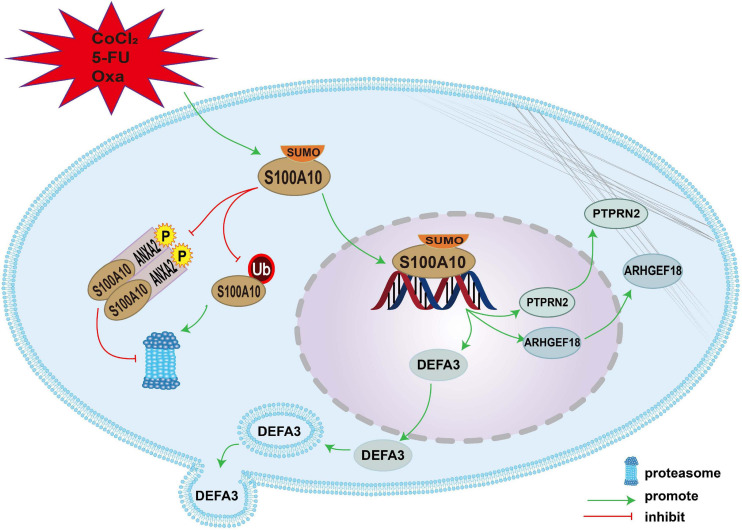
The mechanism of nuclear-located S100A10 regulating the invasion, proliferation and migration of PGCCs with daughter cells. S100A10 was modified by SUMOylation in the PGCCs with daughter cells after CoCl_2_, 5-Fu and Oxa treatment and could not be degraded by proteasome, which resulted in the increase of S100A10 expression in the PGCCs with daughter cells. ANXA2 could form a hereotetramer (AIIt) with S100A10, which could protect S100A10 from degrading by proteasome. Phosphorylation of ANXA2 at tyrosine 23 could promote the formation of AIIt. SUMOylation promoted the nuclear location of S100A10 and S100A10 in the nuclear regulated the expression of *DEFA3, PTPRN2*, and *ARHGEF18*, which related to actin dynamics and cytoskeleton remodeling.

S100A10 can be modified by SUMOylation in PGCCs and their daughter cells induced by CoCl_2_ and chemoradiotherapy. SUMO1-modified S100A10 can translocate to the nucleus and regulate the expression of ARHGEF18, PTPRN2, and DEFA3 to promote the proliferation, migration, and invasion of PGCCs with daughter cells. The mechanism of nuclear-located S100A10 regulating the invasion, proliferation and migration of PGCCs with daughter cells was interpreted in Figure 7.

## Data Availability Statement

The authors declare that all data supporting the findings of this study are available within thearticle and its Supplementary Material or contact the corresponding author uponreasonable request. The raw data of ChIP-seq was submitted to GEO at: https://www.ncbi.nlm.nih.gov/geo/query/acc.cgi?acc=GSE176231.

## Ethics Statement

All the human tissue samples involved in our study were paraffin-embedded tissues in vitroobtained from the department of pathology of Tianjin Union Medical Center, which did notrequire the written informed consent from the patients. The use of human tissue samples wasapproved by the Hospital Review Board of Tianjin Union Medicine Center and the referencenumber is (2021) Fast Review No. (B09). The confidentiality of patient information wasmaintained.

## Author Contributions

SZ designed the study and interpreted data, contributed to manuscript writing, and approved the manuscript before submission. QZ, KZ, and ZL collected and analyzed data and approved the manuscript before submission. ZL, HZ, and FF collected, analyzed, and interpreted data, and approved the manuscript before submission. MZ collected data, gave constructive comments on the manuscript, and approved the manuscript before submission. All authors contributed to the article and approved the submitted version.

## Conflict of Interest

The authors declare that the research was conducted in the absence of any commercial or financial relationships that could be construed as a potential conflict of interest.

## Abbreviations

PGCCs: polyploid giant cells
CoCl_2_: cobalt chloride
iTRAQ: isobaric tags for relative and absolute quantification
GA: ginkgolic acid
WB: Western blotting
5-Fu: 5-fluorouracil
Oxa: oxaliplatin
SUMO: small ubiquitin-like modifier
CRC: colorectal cancer
DEFA3: neutrophil defensin 3
PTPRN2: receptor-type tyrosine-protein phosphatase N2
ARHGEF18: rho guanine nucleotide exchange factor 18
MMP: matrix metalloproteinase
NC: negative-control
AIIt: a hereotetramer combined with S100A10 and ANXA2
LARC: locally advanced rectal cancer
CRC: colorectal cancer
ANXA2: Annexin A2
t-PA: tissue plasminogen activator
ICC: immunocytochemical
IHC: immunohistochemical
co-IP: co-immunoprecipitation
ChIP: Chromatin immunoprecipitation
GO: gene ontology
KEGG: Kyoto Encyclopedia of Genes and Genomes.
