# Supplementary material for: High Migration and Invasion Ability of PGCCs and Their Daughter Cells Associated With the Nuclear Localization of S100A10 Modified by SUMOylation
Source: Front Cell Dev Biol. 2021 Jul 16;9:696871. doi: 10.3389/fcell.2021.696871 (PMC8322665; doi:10.3389/fcell.2021.696871)
Supplement: Supplementary file 1 [file Table_1.DOCX]

**Supplementary table 1. Antibodies of western blot , ICC/IHC and IP/ChIP.**

| Reagent | Species Specificity | Company | WB | ICC/IHC | | IP/ChIP |
| --- | --- | --- | --- | --- | --- | --- |
| S100A10  S100A10 | Rabbit Polyclonal  Mouse monoclonal | Proteintech  BD Biosciences | 1:1000  - | 1:1000  - | - | |
|  |  |  |  |  | 1:50 | |
| ANXA2 | Rabbit polyclonal | CST | 1:3000 | 1:1000 | - | |
| P-ANXA2 (85.Tyr 24) | Mouse monoclonal | SANTA CRUZ | 1:500 | - | - | |
| SUMO1 | Rabbit polyclonal | CST | 1:1000 | - | - | |
| SUMO2/3 | Rabbit monoclonal | CST | 1:1000 | - | - | |
| Ubiquitin(linkage-specific K48) | Rabbit monoclonal | Abcam | 1:1000 | - | - | |
| PTPRN2 | Rabbit polyclonal | BOSTER | 1:500 | - | - | |
| ARHGEF18 | Rabbit polyclonal | Proteintech | 1:500 | - | - | |
| DEFA1 | Rabbit polyclonal | Proteintech | 1:500 | - | - | |
| β-actin | Mouse monoclonal | Proteintech | 1:1000 | -  - | -  - | |
| GAPDH | Rabbit monoclonal | CST | 1:3000 |  |  |  |
