# Supplementary material for: High Migration and Invasion Ability of PGCCs and Their Daughter Cells Associated With the Nuclear Localization of S100A10 Modified by SUMOylation
Source: Front Cell Dev Biol. 2021 Jul 16;9:696871. doi: 10.3389/fcell.2021.696871 (PMC8322665; doi:10.3389/fcell.2021.696871)
Supplement: Supplementary file 2 [file Table_2.DOCX]

**Supplementary table 2. SUMO1-siRNA interfering sequences.**

| Names | Sense (5ʹ-3ʹ) | Antisense (5ʹ-3ʹ) |
| --- | --- | --- |
| SUMO1-307 | GACAGGGUGUUCCAAUGAATT | UUCAUUGGAACACCCUGUCTT |
| SUMO1-358 | GAGAAUUGCUGAUAAUCAUTT | AUGAUUAUCAGCAAUUCUCTT |
| SUMO1-727 | GGCUUGUGGUGAUAAAUAATT | UUAUUUAUCACCACAAGCCTT |
| SUMO1-GAPDH | UGACCUCAACUACAUGGUUTT | AACCAUGUAGUUGAGGUCATT |
| SUMO1-NC | UUCUCCGAACGUGUCACGUTT | ACGUGACACGUUCGGAGAATT |
