# Supplementary material for: High Migration and Invasion Ability of PGCCs and Their Daughter Cells Associated With the Nuclear Localization of S100A10 Modified by SUMOylation
Source: Front Cell Dev Biol. 2021 Jul 16;9:696871. doi: 10.3389/fcell.2021.696871 (PMC8322665; doi:10.3389/fcell.2021.696871)
Supplement: Supplementary file 3 [file Table_3.DOCX]

**Supplementary table 3. SUMO2-siRNA interfering sequences.**

| Names | Sense (5ʹ-3ʹ) | Antisense (5ʹ-3ʹ) |
| --- | --- | --- |
| SUMO2-275 | GCAUACACCACUUAGUAAATT | UUUACUAAGUGGUGUAUGCTT |
| SUMO2-315 | CGACAGGGAUUGUCAAUGATT | UCAUUGACAAUCCCUGUCGTT |
| SUMO2-498 | GACCAAGAUUACAUUCUCATT | UGAGAAUGUAAUCUUGGUCTT |
| SUMO2-GAPDH | UGACCUCAACUACAUGGUUTT | AACCAUGUAGUUGAGGUCATT |
| SUMO2-NC | UUCUCCGAACGUGUCACGUTT | ACGUGACACGUUCGGAGAATT |
