# Supplementary material for: High Migration and Invasion Ability of PGCCs and Their Daughter Cells Associated With the Nuclear Localization of S100A10 Modified by SUMOylation
Source: Front Cell Dev Biol. 2021 Jul 16;9:696871. doi: 10.3389/fcell.2021.696871 (PMC8322665; doi:10.3389/fcell.2021.696871)
Supplement: Supplementary file 4 [file Table_4.DOCX]

**Supplementary table 4. SUMO3-siRNA interfering sequences.**

| Names | Sense (5ʹ-3ʹ) | Antisense (5ʹ-3ʹ) |
| --- | --- | --- |
| SUMO3-471 | CAAUGAAACUGACACUCCATT | UGGAGUGUCAGUUUCAUUGTT |
| SUMO3-744 | CUGCAGGGAUGAAUCUGUATT | UACAGAUUCAUCCCUGCAGTT |
| SUMO3-814 | GCAAGAUAUUGUGGGUACUTT | AGUACCCACAAUAUCUUGCTT |
| SUMO3-GAPDH | UGACCUCAACUACAUGGUUTT | AACCAUGUAGUUGAGGUCATT |
| SUMO3-NC | UUCUCCGAACGUGUCACGUTT | ACGUGACACGUUCGGAGAATT |
