# Supplementary material for: High Migration and Invasion Ability of PGCCs and Their Daughter Cells Associated With the Nuclear Localization of S100A10 Modified by SUMOylation
Source: Front Cell Dev Biol. 2021 Jul 16;9:696871. doi: 10.3389/fcell.2021.696871 (PMC8322665; doi:10.3389/fcell.2021.696871)
Supplement: Supplementary file 5 [file Table_5.DOCX]

**Supplementary table 5. S100A10-siRNA interfering sequences.**

| Names | Sense (5ʹ-3ʹ) | Antisense (5ʹ-3ʹ) |
| --- | --- | --- |
| S100A10-514 | CUCAAAUGGAACACGCCAUTT | AUGGCGUGUUCCAUUUGAGTT |
| S100A10-682 | CCUGGACCAGUGUAGAGAUTT | AUCUCUACACUGGUCCAGGTT |
| S100A10-740 | CCUCACCAUUGCAUGCAAUTT | AUUGCAUGCAAUGGUGAGGTT |
| S100A10-GAPDH | UGACCUCAACUACAUGGUUTT | AACCAUGUAGUUGAGGUCATT |
| S100A10-NC | UUCUCCGAACGUGUCACGUTT | ACGUGACACGUUCGGAGAATT |
