# Supplementary material for: High Migration and Invasion Ability of PGCCs and Their Daughter Cells Associated With the Nuclear Localization of S100A10 Modified by SUMOylation
Source: Front Cell Dev Biol. 2021 Jul 16;9:696871. doi: 10.3389/fcell.2021.696871 (PMC8322665; doi:10.3389/fcell.2021.696871)
Supplement: Supplementary file 6 [file Table_6.DOCX]

**Supplementary table 6. Primers of real-time PCR.**

| Names | Sense (5ʹ-3ʹ) | Antisense (5ʹ-3ʹ) |
| --- | --- | --- |
| PTPRN2 | GAAGCCCGACCCACAACAAA | CCTCTCACATCCGCCATCCT |
| DEFA3 | TGCAGGAGAACGTCGCTATG | TGAGCAGAAGGTACAGGAGT |
| ARHGEF18 | CAACTCGGACCAATCACAGGA | TGTAAGGGACAGAGAGTCATCAG |
