# Supplementary material for: High Migration and Invasion Ability of PGCCs and Their Daughter Cells Associated With the Nuclear Localization of S100A10 Modified by SUMOylation
Source: Front Cell Dev Biol. 2021 Jul 16;9:696871. doi: 10.3389/fcell.2021.696871 (PMC8322665; doi:10.3389/fcell.2021.696871)
Supplement: Supplementary file 7 [file Data_Sheet_1.doc]

**Supplementary figure legends**

**
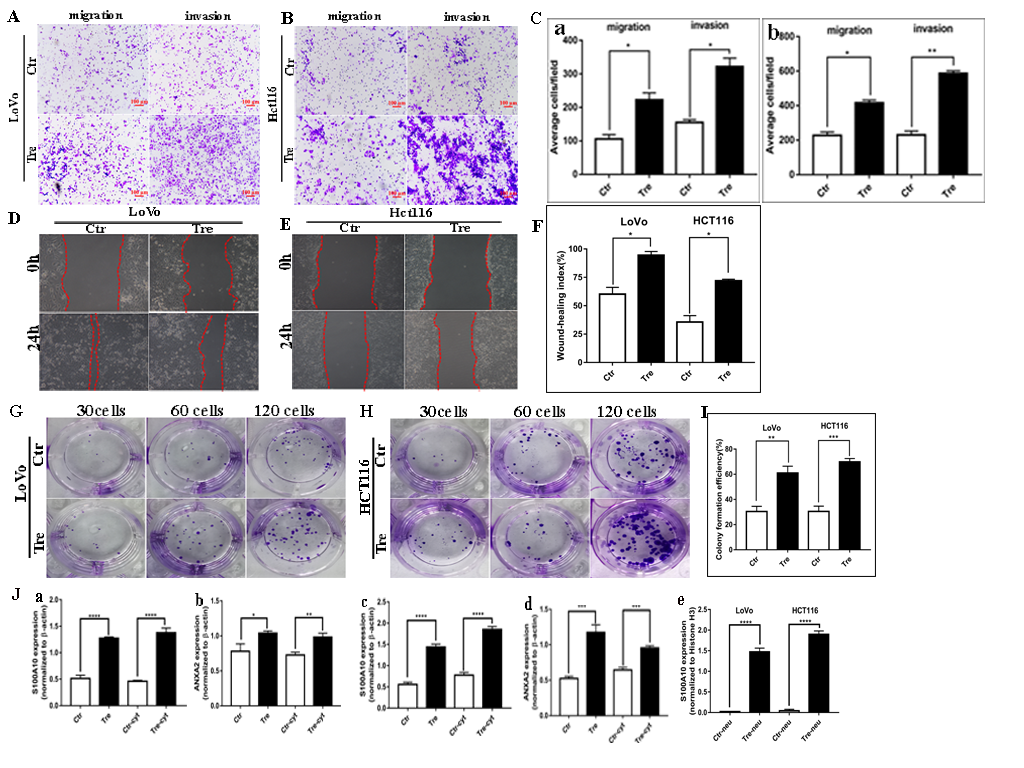
**

**Supplementary figure 1.** **A.** The migration and invasion ability of LoVo cells before and after the treatment with CoCl_2_. **B.** The migration and invasion ability of Hct116 cells before and after the treatment with CoCl_2_. **C.** (a) Comparison of the average cell number in migration and invasion assay of LoVo cells before and after the treatment of CoCl_2_. (b) Comparison of the average cell number in migration and invasion assay of Hct116 cells before and after the treatment of CoCl_2_. **D.** Wound-healing assay of LoVo cells before and after the treatment of CoCl_2_. **E.** Wound-healing assay of Hct116 cells before and after the treatment with CoCl_2_. **F.** Wound-healing index of LoVo and Hct116 cells before and after the treatment with CoCl_2_. **G.** Colony formation of LoVo cells before and after the treatment with CoCl_2_. **H.** Colony formation of Hct116 cells before and after the treatment with CoCl_2_. **I.** Colony formation efficiency of LoVo and Hct116 cells before and after the treatment with CoCl_2_. **J.** (a) Comparison of the total and cytoplasmic S100A10 expression in LoVo cells before and after CoCl2 treatment. (b) Comparison of the total and cytoplasmic expression of ANXA2 in LoVo cells before and after CoCl2 treatment. (c) Comparison of the total and cytoplasmic S100A10 expression in Hct116 cells before and after CoCl2 treatment. (d) Comparison of the total and cytoplasmic ANXA2 expression in Hct116 cells before and after CoCl2 treatment. (e) Comparison of the nuclear S100A10 expression in Hct116 and LoVo cells before and after CoCl2 treatment.


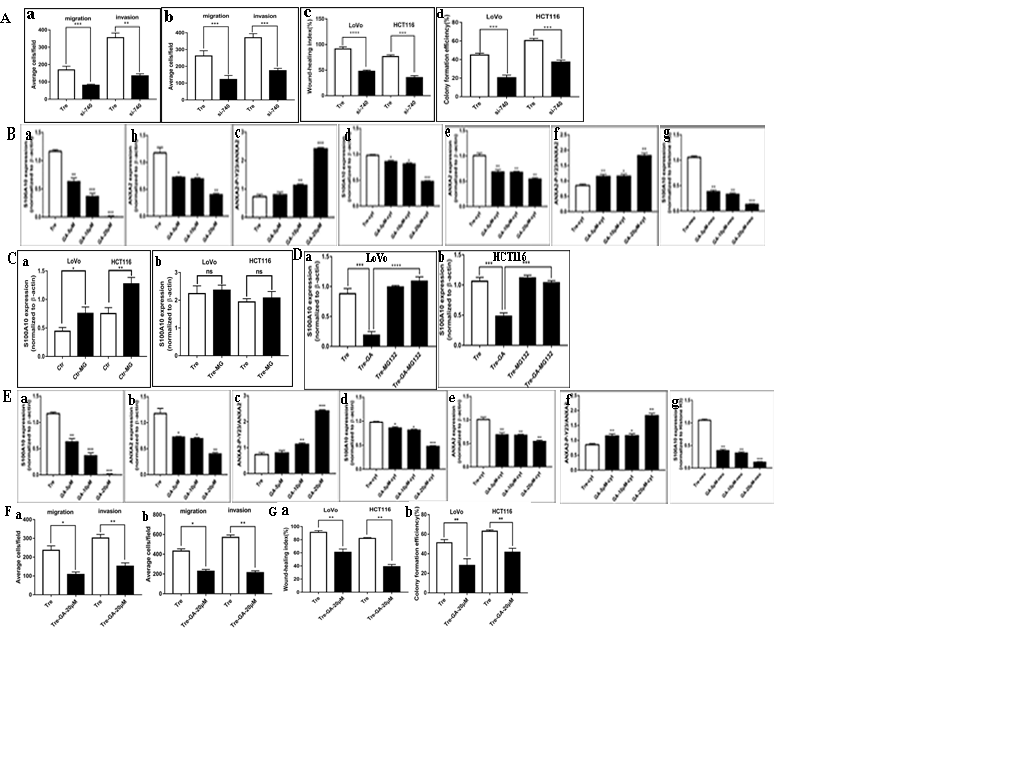


**Supplementary figure 2.** **A.** (a) Comparison of the average cell number for migration and invasion assay in LoVo PGCCs with daughter cells transfected with siRNA S100A10-740 and siRNA control. (b) Comparison of the average cell number for migration and invasion assay in Hct116 PGCCs with daughter cells transfected with siRNA S100A10-740 and siRNA control. (c) Wound-healing index of LoVo and Hct116 PGCCs with daughter cells transfected with siRNA S100A10-740 and siRNA control. (d) Colony formation efficiency of LoVo and Hct116 PGCCs with daughter cells transfected with siRNA S100A10-740 and siRNA control. **B.** Comparison of different protein expression in LoVo PGCCs with daughter cells after ginkgolic acid treatment at 0, 5, 10 and 20 μM, respectively for 24 h. (a) Total S100A10. (b) Total ANXA2. (c) Total ANXA2-P-Y23. (d) Cytoplasmic S100A10. (e) Cytoplasmic ANXA2. (f) Cytoplasmic ANXA2-P-Y23. (g) Nuclear S100A10. **C**. (a) Comparison of S100A10 in LoVo and Hct116 control cells before and after MG132 treatment. (b) Comparison of S100A10 in LoVo and Hct116 PGCCs and daughter cells before and after MG132 treatment. **D**. (a) Comparison of S100A10 in LoVo PGCCs and daughter cells with non-treatment, treatment of GA, treatment of MG132 and treatment of both GA and MG132 respectively. (b) Comparison of S100A10 in Hct116 PGCCs and daughter cells with and without GA, MG132 and both GA and MG132 treatment. **E.** Comparison of different proteins expression in Hct116 PGCCs with daughter cells after the treatment with ginkgolic acid at 0, 5, 10 and 20 μM for 24 h. (a) Total S100A10. (b) Total ANXA2. (c) Total ANXA2-P-Y23. (d) Cytoplasmic S100A10. (e) Cytoplasmic ANXA2. (f) Cytoplasmic ANXA2-P-Y23. (g) Nuclear S100A10. **F.** (a) Comparison of the average cell number for migration and invasion assay in LoVo PGCCs and their daughter cells before and after 20 μM GA treatment for 24 h. (b) Comparison of the average cell number for migration and invasion assay in HCT116 PGCCs and their daughter cells before and after 20 μM GA treatment for 24 h. **G.** (a) Wound-healing index of LoVo and HCT116 PGCCs and their daughter cells before and after 20 μM GA treatment for 24 h. (b) Colony formation efficiency of LoVo and HCT116 PGCCs and their daughter cells before and after 20 μM GA treatment for 24 h.

Ctr: control cells; Tre: cells treated with CoCl_2_; GA: ginkgolic acid treatment

**
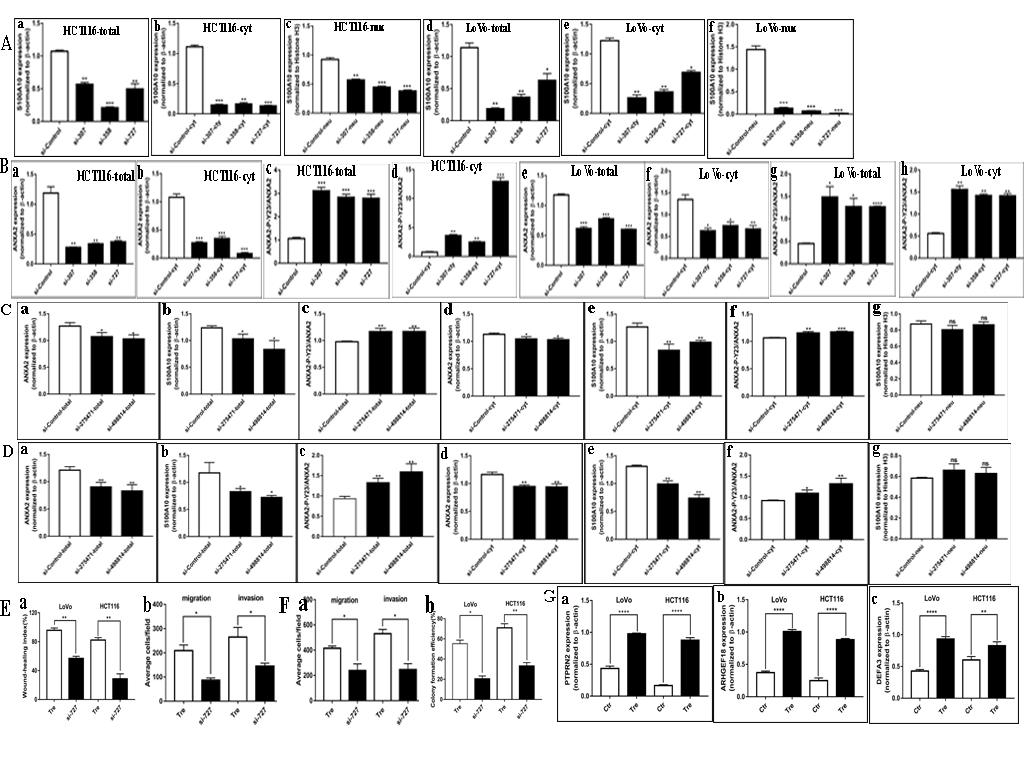
**

**Supplementary Figure 3 :** **A**. Comparison of total S100A10 expression after transfection with siRNA SUMO1-307, 358, 727 and siRNA control. (a) Total S100A10 expression in Hct116 PGCCs and their daughter cells. (b) Cytoplasmic S100A10 expression in Hct116 PGCCs and their daughter cells. (c) Nuclear S100A10 expression in Hct116 PGCCs and their daughter cells. (d) Total S100A10 expression in LoVo PGCCs and their daughter cells. (e) Cytoplasmic S100A10 expression in LoVo PGCCs with their daughter cells. (f) Nuclear S100A10 expression in LoVo PGCCs with their daughter cells. **B**. Comparison of expression of ANXA2 and ANXA2-P-Y23 after transfection with siRNA SUMO1-307, 358, 727 and siRNA control (a) Total expression of ANXA2 in Hct116 PGCCs with their daughter cells. (b) Cytoplasmic expression of ANXA2 in Hct116 PGCCs with their daughter cells. (c) Total expression of ANXA2-P-Y23 in Hct116 PGCCs with their daughter cells. (d) Cytoplasmic expression of ANXA2-P-Y23 in Hct116 PGCCs with their daughter cells. (e) Total expression of ANXA2 in LoVo PGCCs with their daughter cells. (f) Cytoplasmic expression of ANXA2 in LoVo PGCCs with their daughter cells. (g) Total expression of ANXA2-P-Y23 in LoVo PGCCs with their daughter cells. (h) Cytoplasmic expression of ANXA2-P-Y23 in LoVo PGCCs with their daughter cells. **C.** Comparison of S100A10 and S100A10 related proteins expression after transfection with siRNA SUMO2/3 and siRNA control in LoVo PGCCs and their daughter cells. (a) Total expression of ANXA2 (b) Total expression of S100A10 (c) Total expression of ANXA2-P-Y23 (d) Cytoplasmic expression of ANXA2. (e) Cytoplasmic expression of S100A10. (f) Cytoplasmic expression of ANXA2-P-Y23.(g) Nuclear expression of S100A10. **D.** Comparison of S100A10 and S100A10 related proteins expression after transfection with siRNA SUMO2/3 and siRNA control inHCT116 PGCCs and their daughter cells. (a) Total expression of ANXA2 (b) Total expression of S100A10 (c) Total expression of ANXA2-P-Y23 (d) Cytoplasmic expression of ANXA2. (e) Cytoplasmic expression of S100A10. (f) Cytoplasmic expression of ANXA2-P-Y23.(g) Nuclear expression of S100A10. **E.** (a) Wound-healing index of LoVo and Hct116 PGCCs with daughter cells transfected with SUMO1-727 and control siRNAs. (b) Comparison of the average cell number for migration and invasion assay in LoVo PGCCs with daughter cells transfected with SUMO1-727 and control siRNAs. **F.** (a) Comparison of the average cell number for migration and invasion assay in Hct116 PGCCs with daughter cells transfected with SUMO1-727 and control siRNAs. (b) Colony formation efficiency in LoVo and Hct116 PGCCs with daughter cells transfected with SUMO1-727 and control siRNAs. **G.** Comparison of different proteins expression in LoVo and Hct116 cells before and after CoCl_2_ treatment. (a) Total PTPRN2. (b)Total ARHGEF18. (c)Total DEFA3.

**
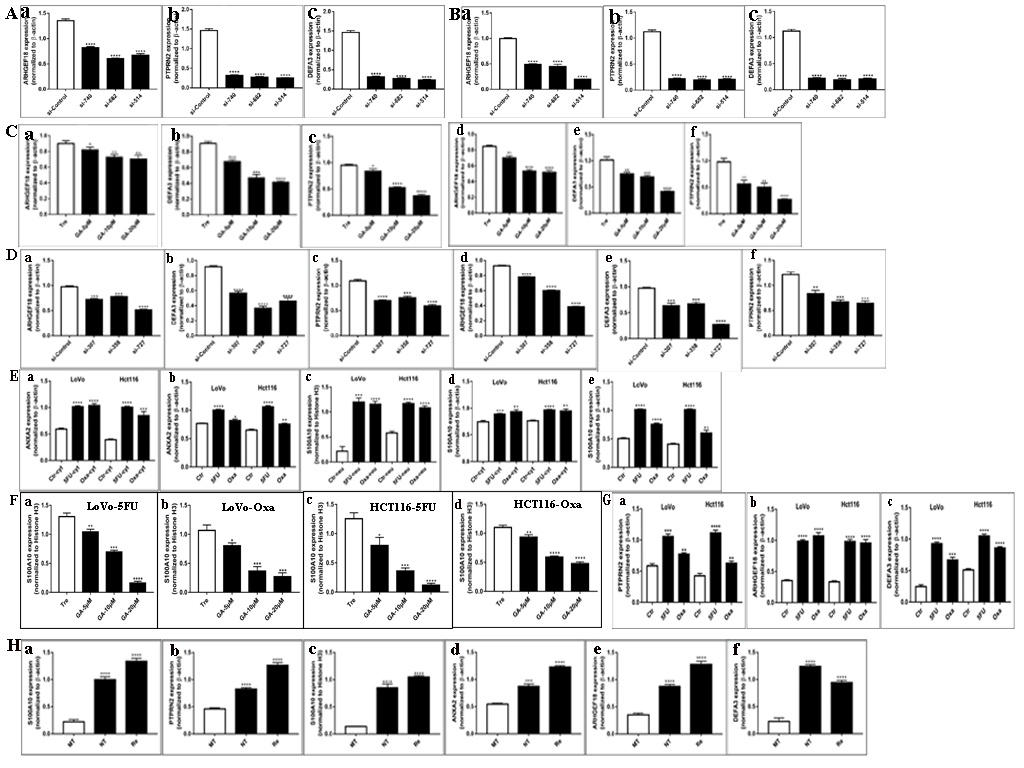
**

**Supplementary figure 4. A.** Comparison of different proteins expression in LoVo PGCCs and daughter cells transfected with siRNA S100A10-740, 682, 514, and siRNA control. (a) Total ARHGEF18. (b) Total PTPRN2. (c) Total DEFA3. **B.** Comparison of different proteins expression in Hct116 PGCCs and daughter cells transfected with siRNA S100A10-740, 682, 514, and siRNA control. (a) Total ARHGEF18. (b) Total PTPRN2. (c) Total DEFA3. **C.** Comparison of different protein expression in LoVo and HCT116 PGCCs with daughter cells after ginkgolic acid treatment at 0, 5, 10 and 20 μM, respectively for 24 h. (a)Total ARHGEF18 of LoVo (b) Total DEFA3 of LoVo (c) Total PTPRN2 of LoVo (d)Total ARHGEF18 of HCT116 (e) Total DEFA3 of HCT116 (f) Total PTPRN2 of HCT116. **D.** Comparison of different proteins expression in LoVo PGCCs and daughter cells transfected with siRNA SUMO1-307, 358, 727, and siRNA control. (a) Total ARHGEF18. (b) Total DEFA3. (c) Total PTPRN2. (d) Total ARHGEF18. (e) Total DEFA3. (f) Total PTPRN2. **E.** Comparison of different proteins expression in LoVo and Hct116 PGCCs with daughter cells after 5-FU and Oxa treatment. (a) Total ANXA2. (b) Cytoplasmic ANXA2. (c) Nuclear S100A10. (d). Total S100A10. (e) Cytoplasmic S100A10. **F.** (a) Comparison of nuclear protein expression of S100A10 in 5FU-induced LoVo PGCCs with daughter cells after GA treatment at 0, 5, 10 and 20 μM, respectively for 24 h. (b) Comparison of nuclear protein expression of S100A10 in Oxa-induced LoVo PGCCs with daughter cells after GA treatment at 0, 5, 10 and 20 μM, respectively for 24 h. (c) Comparison of nuclear protein expression of S100A10 in 5FU-induced HCT116 PGCCs with daughter cells after GA treatment at 0, 5, 10 and 20 μM, respectively for 24 h. (d) Comparison of nuclear protein expression of S100A10 in Oxa-induced HCT116 PGCCs with daughter cells after GA treatment at 0, 5, 10 and 20 μM, respectively for 24 h. **G.** Comparison of different proteins expression in LoVo and Hct116 PGCCs with daughter cells after 5-FU and Oxa treatment. (a) Total PTPRN2. (b) Total ARHGEF18. (c) Total DEFA3. **H.** Comparison of different proteins expression in moderately differential CRC tissues, moderately differential CRC tissues with nCRT and recurrent CRC tissues. (a) Cytoplasmic S100A10. (b) Cytoplasmic PTPRN2. (c) Nuclear S100A10. (d) Cytoplasmic ANXA2. (e) Cytoplasmic ARHGEF18. (f) Cytoplasmic DEFA3.
