# Supplementary material for: High Migration and Invasion Ability of PGCCs and Their Daughter Cells Associated With the Nuclear Localization of S100A10 Modified by SUMOylation
Source: Front Cell Dev Biol. 2021 Jul 16;9:696871. doi: 10.3389/fcell.2021.696871 (PMC8322665; doi:10.3389/fcell.2021.696871)
Supplement: Supplementary file 8 [file Data_Sheet_2.docx]

**Supplementary materials and methods**

**1. Cell lines and culture conditions**

Hct116 and LoVo CRC cells were obtained from the American Type Culture Collection and grown in complete RPMI-1640 (Gibco, Thermo Fisher Scientific, Suzhou, China) medium supplemented with 10% fetal bovine serum (FBS; Gibco, Life Technologies, New Zealand), 100 U/mL penicillin, and 100 μg/mL streptomycin (Gibco, Life Technologies, USA). The cells were maintained at 37°C in a humidified atmosphere containing 5% CO_2_.

**2. Induction of PGCCs formation using CoCl_2_ treatment**

LoVo and Hct116 cells were incubated in T25 flasks in RPMI-1640 medium until they reached 80-90% confluence. The cells were treated with 450 μM CoCl_2_ (Sigma-Aldrich, St. Louis, MO, USA) for 48-72 h based on their resistance to hypoxia. The detail treatment method has been described in our previously published studies [[2](#_ENREF_2)]. After the treatment with CoCl_2_ was repeated for 3-4 times, there were 20-30% of PGCCs and 70-80% of small-sized daughter cells that originated from PGCCs among the total cells. PGCCs were defined as multinuclear cancer cells and were three times larger in size than regular cancer cells with smaller size, which served as control cells.

**3. 5-fluorouracil and Oxaliplatin treatment**

LoVo and Hct116 cells were cultured in T25 flasks until they reached 80-90% confluence, and 5-Fu 150 μM for 48 h and Oxa (LoVo: 2 μM for 48 h; Hct116: 5 μM for 48 h) were added into the flasks. After treatment, the medium was changed and the cells were cultured in medium without 5-Fu and Oxa. Most of the small-size cells died, and only a few giant PGCCs survived the 5-Fu and Oxa treatment. One week later, the surviving PGCCs recovered from the treatment and began to produce daughter cells via asymmetric division. When the confluence of PGCCs with their daughter cells reached 90%, the cells were treated with 5-Fu and Oxa for the second time. The number of surviving PGCCs was higher than that after the first treatment. After repeating the treatment with 5-Fu and Oxa for 3-4 times, there were 20-30% PGCCs and 70-80% daughter cells in the flask, and these cell samples were collected for further experiments.

**4. Western blots analysis**

CoCl_2_ treated and untreated cells were collected. Total, cytoplasmic, and nuclear proteins were extracted according to the manufacturer’s instructions (Thermo Fisher Scientific; #SJ252790). The total protein amount was quantified using a BCA Protein Assay Kit (Thermo Fisher Scientific; #23225), and then the proteins were separated using a 12.5% sodium dodecyl sulfate polyacrylamide gels and transferred to polyvinylidene fluoride membranes (Beyotime, Hamen, China). The membranes were blocked using 5% skim milk at room temperature for 2 h and then detected with the different primary antibodies. The detailed information of the antibodies used is listed in Supplementary Table 1. The membranes were then incubated with the appropriate secondary antibodies for 2 h at room temperature. The images were obtained using a ChemiDoc imaging system (BioRad, USA). β-actin, GAPDH, and histone H3 were used as protein loading controls. All WB analyses were repeated three times independently.

**5. Cell migration and invasion assays**

Wound-healing and transwell migration assays were used to detect cell migration ability. Cells were cultured in 6-well plates until they reached 95% confluency, and then the surfaces of the plates were scratched using sterile pipette tips to create wounds. Phosphate-buffered saline was used to wash away the detached cells. Then, the cells were incubated in serum-free RPMI 1640 medium. Images of the closing wounds were captured at 0, 12, and 22 h. The scratched area was measured using ImageJ software.

For transwell migration assays, the cell culture inserts (8 μm, BD-Falcon, Franklin Lakes, NJ, USA) were placed into 24-well plates. The cells (10×10^4^ cells per insert) in 200 μL medium without FBS were added to the upper chambers, and the medium supplemented with 20% FBS was added to the lower chamber. After 24 h of incubation, cell culture inserts were fixed with cold methanol for 30 min and stained with 0.1% crystal violet for 30 min. Images were captured at 100× magnification, and the average number of stained cells was counted in at least five different fields.

Cell invasion ability was determined using the transwell invasion assay, and each insert was precoated with BD Matrigel Basement Membrane Matrix (Corning). Cell suspensions containing 5×10^5^ cells and 200 μL of medium without FBS were cultured in the upper chamber. The bottom chamber contained 600 μL of the medium with 20% FBS. The cells were fixed with cold methanol for 30 min and stained with 0.1% crystal violet for 30 min after incubation for 22 h. The invasive cells were counted and imaged at 100× magnification. The higher number of the stained invasive cells corresponded to the stronger cell invasive ability.

**6. Colony formation assay**

Cell samples were diluted to 30, 60, and 120 cells/mL and then cultured in 12-well plates. The plates were incubated for 1-2 weeks. When the colonies could be observed, the plates were fixed with cold methanol for 30 min and stained with 0.1% crystal violet for 30 min. Cell colonies were counted at 100× magnification (a single colony was defined as one containing more than 50 cells). Cell colony formation efficiency was assessed using the following formula: formation efficiency = number of clones/number of cells inoculated.

**7. Co-immunoprecipitation**

Co-IP was used to determine the interactions of SUMO1-S100A10 and ubiquitin-S100A10 in LoVo and Hct116 cells before and after CoCl_2_ treatment. The cells were lysed using IP lysis buffer (Thermo Fisher Scientific) containing halt protease and phosphatase inhibitor cocktail (1:100 dilution) for 30 min on ice and then centrifuged at 14000 rpm for 10 min. The supernatant was collected and incubated with mouse anti-S100A10 monoclonal antibody overnight at 4°C on a roller. Normal mouse IgG (Beyotime, Shanghai, China) was used as a negative control. The pre-washed protein A/G agarose beads (Thermo Fisher Scientific) were added to each IP tube and incubated for 2 h at 4°C. After washing and centrifugation, the IP results were confirmed by WB using an anti-S100A10 monoclonal antibody. IP samples were also immunoblotted with anti-SUMO1 and anti-ubiquitin (linkage-specific K48) to detect the SUMO- or Ubi-modifications of S100A10.

**8. Cell viability assay**

GA (15:1) is an in vitro small-molecule inhibitor of SUMOylation. It can directly bind to SUMO E1 activating enzyme and inhibit the formation of the E1-SUMO intermediate. To assess the cell viability before and after GA (15:1, MedChem Express, USA) treatment, LoVo and Hct116 PGCCs with daughter cells were seeded at 5,000 cells per well in 96-well plates and incubated for 12 h. These cells were divided into five groups, and each group was independently repeated in triplicate. The cells were treated with GA at concentrations of 0, 5, 10, 20, and 40 μM for 12, 24, 36, and 48 h, respectively. After incubation, 10 µL of CCK8 (Dojndo, Japan) was added to each well and incubated for 2 h, including three control wells containing medium alone, which were used as blanks for absorbance readings. These wells were detected using a Bio-Rad microplate reader at a wavelength of 450 nm. The optical density data were presented as the means±standard errors.

**9.Immunocytochemical and immunohistochemical staining**

The cells were cultured on the cover slips and fixed with cold methanol for 30 min at room temperature when the confluency reached 70-80%. Next, the cells were incubated with 0.3% endogenous peroxidase inhibitor (Zhongshan Inc., Beijing, China) for 15 min and then blocked with 1.5% normal goat serum (Zhongshan Inc., Beijing, China) for 20 min. The primary antibodies used for ICC and immunohistochemical (IHC) are listed in Supplementary Table 1. After incubation with primary antibodies at 4ºC overnight, biotinylated goat anti-mouse/rabbit IgG (Zhongshan Inc., Beijing, China) and horseradish peroxidase-labeled streptomycin (Zhongshan Inc., Beijing, China) were added to the slides for 20 and 15 min, respectively. Detailed ICC analysis was performed according to the instructions provided in the Biotin-Streptavidin HRP Detection Systems (SP-9000, Zhongshan Inc.). For IHC staining, paraffin-embedded tissue samples were baked at 70ºC for 2 h and deparaffined in xylene. A graded series of different alcohol concentrations were used to dehydrate paraffin-embedded tissue sections. The sections were subjected to antigen retrieval in 0.01 M sodium citrate buffer (pH 6.0) in an autoclave for 1-2 min, and the endogenous peroxidase activity was blocked using 0.3% hydrogen peroxide. After the sections were incubated with primary antibodies and underwent reaction with biotinylated goat anti-rabbit IgG antibody for 20 min, the signal was detected using the labeled streptavidin-biotin system in the presence of the chromogen 3,3-diaminobenzidine or alkaline phosphatase. Nuclei were counterstained using hematoxylin.

**10. Transient siRNA transfection**

Three different siRNA interference sequences targeting each SUMO-1 and S100A10, and negative control siRNA oligonucleotides were obtained from Gene-Pharma (Shanghai, China). Transfection was performed when cells reached 60-70% confluence in 6-well plates. Negative control siRNA, SUMO1-siRNA, and S100A10-siRNA were diluted with Lipofectamine 2000 (Invitrogen, Carlsbad, CA, USA) and 1×Opti-MEM (Gibco, USA) according to the manufacturer’s protocol and added to cells. After 48 h of transfection, cell samples were collected to detect the inhibition efficiency of the targeted protein using WB. Detailed information about the siRNA oligonucleotide sequences is provided in Supplementary Tables 2 and 3.

**11. Chromatin immunoprecipitation and data analysis**

ChIP assays were performed according to the instructions of the Pierce Magnetic ChIP Kit (Thermo Fisher Scientific, #26157) with little modification. Cell samples (10^7^ cells/sample) were crosslinked using 1% formaldehyde for 10 min. Glycine solution (10×) was added to each sample containing cell culture media and formaldehyde to a final concentration of 1×. The samples were mixed well and incubated at room temperature for 5 min. Diluted micrococcal nuclease was used to digest the DNA, and the nuclear membrane was broken using Scientz-ⅡD (10% power, 4 s on and 9 s off) for 2 min. After centrifugation, the supernatant was subjected to immunoprecipitation with anti-RNA polymerase II antibody, normal mouse IgG, and anti-S100A10 monoclonal antibody at 4°C overnight. After IP elution and DNA recovery, the samples were collected for sequencing and real-time PCR analysis. For data analysis of ChIP DNA, sequencing and library preparation were performed using Novogene Technology Co., Ltd. (Tianjin, China). Raw data (raw reads) of FASTQ format were first processed through in-house PERL scripts, and then reference genome and gene model annotation files were downloaded directly from the genome website. For a specific ChIP-seq binding site, individual reads were mapped to the plus or minus strand and presented significant enrichment. After mapping the reads to the reference genome, the MACS2 version 2.1.0 (model-based analysis of ChIP-seq) peak finding algorithm was used to identify regions of specific IP enrichment over background. A p-value threshold of enrichment of 0.05 was used for all data sets. The interaction between transcription factor or chromatin histone modification and DNA was not random, but they showed some specific sequence preference. MEME [^52^](#_ENREF_52) and DREME [^53^](#_ENREF_53) were used to detect the sequence motif, which was used to detect long and short consensus sequences. The position of peak summit around the transcript start sites of genes can predict the interaction sites of proteins and genes. Genes associated with different peaks were identified, and GO and KEGG enrichment analyses were performed.

**12. Real-time PCR analysis**

DNA samples obtained from ChIP were detected using real-time quantitative PCR. For Taqman PCR, each reaction contained 2 μL of DNA diluted in nuclease free water, 25 μL of Universal PCR Master Mix (CWBIO, 0957), and 1 μL of 10 μM forward primer to which 1 μL of 10 μM reverse primer and nuclease-free water wer added to a final volume of 50 μL. Amplification and detection were performed using the ABI Prism 7500 sequencer. The detection system (PE Applied Biosystems) and the thermal cycle program used in this study were: 10 min at 95°C, followed by 40 cycles of 15 s denaturation at 95°C and 1 min annealing at 60°C. ARGHEF18, DEFA3, and PTPRN2 DNA levels were detected using a set of primers. The sequences of PCR primers are shown in Supplemental Table 4.

**13. Scoring of IHC Staining**

Protein expression was evaluated as previously described ^[51](#_ENREF_51)^. Yellow staining in the cytoplasm and/or nucleus was considered positive. The staining intensity and percentage of positive cells were evaluated. The staining intensity was scored as: 0, negative (no staining); 1, weak positive (light yellow staining); 2, moderately positive (brownish yellow staining); and 3, strongly positive (brown staining). The percentage of positive cells was defined as: 0 (negative): <5% positive cells; 1 (weak positive): 6-30 % positive cells; 2 (moderate positive): 31-50% positive cells; and 3 (strong positive): 51-100% positive cells. For S100A10 nuclear staining, 0 was defined as (–), 1 and 2 were defined (+), and 3 was defined (+ +). The staining index for each section was determined by the sum of the staining intensity and positive cell scores.
